# Mimicking immune signatures of flavivirus infection with targeted adjuvants improves dengue subunit vaccine immunogenicity

**DOI:** 10.1038/s41541-019-0119-3

**Published:** 2019-06-25

**Authors:** Katell Bidet, Victor Ho, Collins Wenhan Chu, Ahmad Nazri Mohamed Naim, Khaing Thazin, Kuan Rong Chan, Jenny G. H. Low, Milly M. Choy, Lan Hiong Wong, Paola Florez de Sessions, Yie Hou Lee, Martin L. Hibberd, Eng Eong Ooi, Katja Fink, Jianzhu Chen

**Affiliations:** 1 Interdisciplinary Research Group in Infectious Diseases, Singapore-MIT Alliance for Research and Technology, Singapore, Singapore; 2 Singapore Immunology Network, Agency for Science, Technology and Research, Singapore, Singapore; 3 School of Biological Sciences, Nanyang Technological University, Singapore, Singapore; 4 Genome Institute of Singapore, Agency for Science, Technology and Research, Singapore, Singapore; 5 Department of Biological Sciences, National University of Singapore, Singapore, Singapore; 6 Emerging Infectious Diseases, Duke-NUS Graduate Medical School, Singapore, Singapore; 7 Department of Infectious Diseases, Singapore General Hospital, Singapore, Singapore; 8 KK Women’s and Children’s Hospital, Singapore, Singapore; 9 Department of Pathogen Molecular Biology, London School of Hygiene and Tropical Medicine, London, WC1E 7HT UK; 10 Koch Institute for Integrative Cancer Research and Department of Biology, Massachusetts Institute of Technology, Cambridge, MA 02139 USA

**Keywords:** Adjuvants, Immunology

## Abstract

Neutralizing antibodies (nAbs) are a critical component for protection against dengue virus (DENV) infection, but little is known about the immune mechanisms governing their induction and whether such mechanisms can be harnessed for vaccine development. In this study, we profiled the early immune responses to flaviviruses in human peripheral blood mononuclear cells and screened a panel of toll-like receptor (TLR) agonists that stimulate the same immune signatures. Monocyte/macrophage-driven inflammatory responses and interferon responses were characteristics of flavivirus infection and associated with induction of nAbs in humans immunized with the yellow fever vaccine YF-17D. The signatures were best reproduced by the combination of TLR agonists Pam_3_CSK_4_ and PolyI:C (PP). Immunization of both mice and macaques with a poorly immunogenic recombinant DENV-2 envelope domain III (EDIII) induced more consistent nAb and CD4^+^ T-cell responses with PP compared to alum plus monophosphoryl lipid A. Induction of nAbs by PP required interferon-mediated signals in macrophages in mice. However, EDIII + PP vaccination only provided partial protection against viral challenge. These results provide insights into mechanisms underlying nAb induction and a basis for further improving antigen/adjuvant combinations for dengue vaccine development.

## Dengue vaccine: adjuvants for induction of neutralizing antibodies

Neutralizing antibodies (nAb) are associated with protection from symptomatic dengue virus infection and current dengue vaccine development aims to elicit a strong nAb response. However, immune mechanisms underlying nAb development in response to dengue infection or vaccination are not completely understood. In this study, led by Jianzhu Chen from the Interdisciplinary Research Group in Infectious Diseases in Singapore, researchers identify a combination of two TLR agonists that elicits better nAb responses than currently used adjuvants. Comparison of transcriptomes of human PBMCs infected with flaviviruses identifies an immune signature that is associated with induction of nAbs and a similar immune signature can be elicited with a combination of two TLR agonists. Vaccination of mice or non-human primates using these two TLR agonists as adjuvant elicits a strong nAb response. However, this vaccination regimen could not confer protection in the animals, suggesting that further improvements of this vaccine candidate will be necessary.

## Introduction

The dengue viruses (DENV) are single-stranded RNA viruses of the flavivirus family and are divided into four serotypes (DENV-1-4).^1^ DENV infection is initiated by bite of an infected mosquito. In the skin, Langerhans cells, conventional dendritic cells (DCs), macrophages (Mφs) and keratinocytes are believed to be the primary targets of infection, which can then spread to other organs through draining lymph nodes.^2–4^ In most individuals, DENV infection induces virus-specific Abs and CD4^+^ and CD8^+^ T cells, which provide protection against subsequent infection by the same serotype but only temporary cross-protection against different serotypes of DENV.^1,4^

Neutralizing antibodies (nAbs), which bind to viral structures and prevent virus infection by blocking virus binding to and/or fusion with the host cells,^5–7^ are a critical component in the protection against DENV. Epidemiological studies have shown that high titers of pre-existing nAb (up to >1:300) are associated with lower disease incidence.^8–10^ nAbs are also required for protection conferred by the most effective flavivirus vaccine developed to date, the yellow fever vaccine YF-17D.^11^ Systems biology studies of the YF-17D infection in human subjects have shown that nAb responses are associated with the activation of type I interferon (IFN) responses and could be predicted by the induction of individual transcripts such as the B-cell growth factor receptor TNFRSF17.^12–14^ On the other hand, B-cell and plasmablast (PB) responses were negatively correlated with YF-17D-induced nAb responses in the same cohorts.^13^ Ab responses to DENV are characterized by a dramatic increase in circulating Ab-producing cells or PBs resulting in high titers of DENV-reactive Abs.^15,16^ However, this is not correlated with nAb responses,^17,18^ suggesting that generation and/or selection of nAbs might require immune events different from those mediating the development of high titers of reactive Abs. Despite their importance in dengue vaccine development, the immune mechanisms underlying nAb response to DENV infection are largely unknown.

Induction of strong nAb responses has been at the center of the quest to develop effective dengue vaccines. The only DENV vaccine approved to date, a tetravalent chimeric live attenuated virus (LAV), induces imbalanced and poorly protective responses in DENV-naïve subjects.^19,20^ Subunit vaccines based on recombinant proteins could potentially improve the quality of immune responses, as they offer the possibility to control the epitopes and amount of antigen delivered and to fine-tune the type of immune responses induced using adjuvants. The use of the envelope protein domain III (EDIII), which is the target of highly neutralizing and protective serotype-specific Abs, was investigated as a possible way to circumvent immune interference between serotypes. However, immunogenicity of EDIII was poor and required an extensive immunization schedule and high doses in order to elicit nAb responses.^20^ Therefore, new strategies targeted specifically at increasing nAb responses are needed.

It has become increasingly clear that different vaccines rely on the activation of specific immune programs that determine immunogenicity.^12,13,21^ Differences in the activation of innate immune receptors—including toll-like receptors (TLR)—after a natural infection can tailor the generation of adaptive immune responses specific to the pathogen.^22,23^ These differences could be harnessed for vaccine development, where the use of specific combinations of TLR agonists led to optimization of responses against several pathogens.^24–26^

In this study, we sought to identify immune signatures associated with induction of high nAb responses to DENV in humans and mimic these mechanisms with targeted adjuvants to enhance nAb responses to a model DENV subunit vaccine. We applied a systems biology approach to identify the immune signatures following infection of human PBMCs with a panel of clinically well-characterized flaviviruses. We showed that IFN responses and proinflammatory responses driven by monocytes/Mφs are consistently associated with infection by immunogenic flaviviruses. We reproduced these signatures using a selected combination of two TLR agonists. Immunization of both mice and macaques with the recombinant EDIII in the presence of the adjuvant combination induced more consistent nAb and T-cell responses, as well as partial protection against virus challenge. Our study identifies the early immune signatures associated with nAb responses to DENV and adjuvant combinations that mimic such immune signatures to promote more consistent immune responses to DENV subunit vaccine among recipients.

## Results

### Flaviviruses and adjuvants induce PBMC responses in vitro

**Fig. 1:**
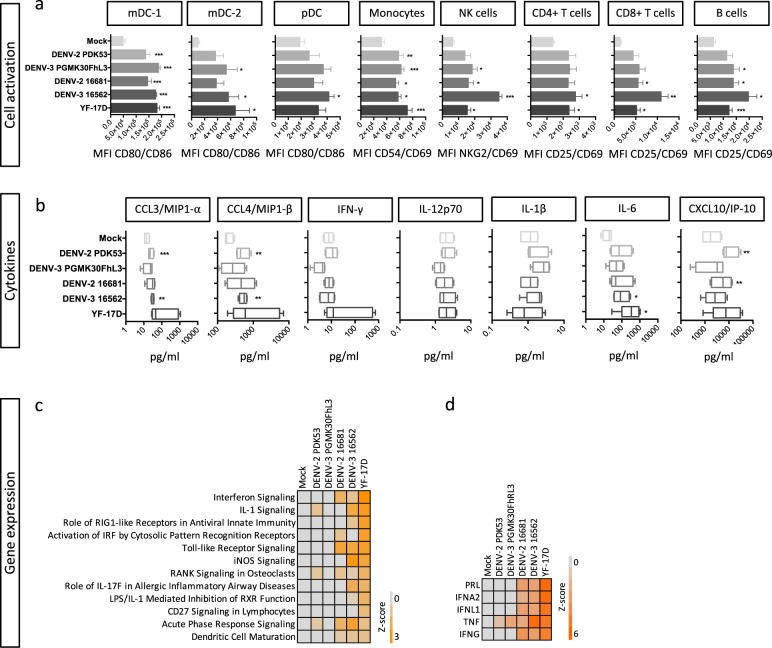
Immune signatures of PBMC responses to flavivirus infection. PBMCs from four healthy donors were infected with the different viruses at MOI = 1 and analyzed after 48 h for immune cell activation, cytokine secretion, and transcriptomic response. **a** MFI of activation markers on immune cell subtypes. Antibodies against the two activation markers were labeled with the same fluorophore. **b** Cytokine concentrations in the supernatant, in pg/ml. Data shown are box plots depicting the median, 25th and 75th percentiles with whiskers showing the min to max distribution of four independent donors. Significant differences are determined using a two-tailed paired *t* test between infected and mock condition for each donor. *p < 0.05, **p < 0.01, ***p < 0.005. **c**, **d** Heatmap of *Z*-score of pathways upregulated after infection (**c**) and upstream regulators (**d**) analyzed by ingenuity pathway analysis

To investigate which adjuvants could mimic features of immunogenic virus infections, we used human peripheral blood mononuclear cells (PBMCs) as a model system. We validated the system by comparing the transcriptional changes in four healthy donor PBMCs following YF-17D infection in vitro to the changes in whole blood of four volunteers after YF-17D vaccination (Supplementary Fig. 1). Consistent with previous reports, IFN signatures dominated both the in vivo and in vitro responses to infection. In total 94 out of 173 differentially expressed transcripts and 9 out of 10 top upstream regulators identified by ingenuity pathway analysis (IPA) changed by in vivo infection were similarly changed after in vitro infection. As observed in vaccinees,^12^ YF-17D infection of PBMCs also induced upregulation of activation markers on various immune cells, including CD80 and CD86 on myeloid DCs (mDCs) and CD54 and CD69 on monocytes (Fig. 1a and Supplementary Fig. 2). These results suggest that the PBMC model system could be a useful alternative to in vivo experiments to study the signatures of early response to flaviviruses.

To identify immune signatures induced by viral infection we compared a panel of five well-characterized flaviviruses in the PBMC model system. Four viruses were from dengue vaccine development efforts at Mahidol University: the LAVs DENV-2 PDK53 and DENV-3 PGMK30FhL3 and their parent viruses DENV-2 16681 and DENV-3 16562, isolated from dengue hemorrhagic fever patients. In tetravalent formulations in clinical trials, DENV-3 PGMK30FhL3 was consistently more immunogenic than DENV-2 PDK53 in inducing both seroconversion and T-cell responses.^27–30^ The fifth virus was the LAV YF-17D, a benchmark for a safe flavivirus vaccine with reliable induction of high nAb titers and CD8 T-cell responses in most vaccinees.^12^ PBMCs from four healthy, dengue-naïve donors were infected with the viruses and expression of activation markers on mDCs, plasmacytoid DCs, monocytes, NK, T, and B cells was analyzed (Supplementary Fig. 2); cytokines in the culture supernatants were quantified; and the transcriptomic profile of the infected cells was analyzed by microarray after 48 h.

To delineate which immune signatures contribute to strong B-cell and T-cell responses rather than to virus pathogenicity or changes in cell metabolism, we applied the following criteria: signatures of interest should be (1) consistently upregulated or downregulated across several viruses, (2) shared by LAV and WT viruses, and (3) strongly activated after infection by YF-17D. All WT and LAVs induced the upregulation of activation markers CD80 and CD86 on BDCA-1^+^ mDC (mDC-1) and monocytes, but not consistently on other cell types (Fig. 1a). DENV-3 16562 and YF-17D were the only two viruses that induced a significant upregulation of the activation markers CD69 and CD25 on CD4, CD8 T cells and B cells. Although there was more variability in the levels of cytokines induced by the different viruses in different donors, we could detect significant upregulation of CCL3/MIP-1α, CCL4/MIP-1β, IL-6, and CXCL10/IP-10 by two or more viruses (Fig. 1b), consistent with previous reports.^12,31,32^ Transcription of hundreds of genes was significantly altered after infection. DENV-2 16681, DENV-3 16562, and YF-17D induced the most numbers of changes (Supplementary Fig. 3a). Based on IPA analysis, only DENV-2 16681, DENV-3 16562, and YF-17D induced activation of pathways related to innate immune responses (Supplementary Fig. 3b). Among the pathways most strongly activated (with the highest *Z*-score) by YF-17D were IFN signaling, IL-1 signaling, TLR signaling, and acute phase response signaling. DC maturation appeared as an important component of the cellular responses to infection that was shared by all viruses (Fig. 1c). This was consistent with the transcriptional activity of proinflammatory cytokines TNF and IFNs (IFN-α, IFN-γ, and IFN-λ) as upstream regulators (Fig. 1d).

In parallel, we assayed the responses of the same four donor PBMCs to a panel of TLR agonists: TLR1/2 agonist Pam_3_CSK_4_ (triacylated lipopeptide), TLR3 agonist PolyI:C (synthetic double-stranded RNA), TLR4 agonist LPS (bacterial lipopolysaccharide), TLR7/8 agonist R848 (nucleoside analog), and TLR9 agonist ODN2006 (CpG oligonucleotide). Aluminum hydroxide (alum), a traditional adjuvant used in many commercial vaccines, served as a non-TLR agonist control. Compared to alum, which did not activate any immune cells, the TLR agonists activated many cell types and all five agonists activated mDC-1 (Supplementary Fig. 4a). All stimuli significantly induced IL-6 and IL-1β secretion (Supplementary Fig. 4b). Pam_3_CSK_4_, LPS, and R848 also stimulated secretion of CCL3/MIP-1α, CCL4/MIP-1β, and IFN-γ. Based on the transcriptomic profile, alum induced signatures of translational downregulation similar to DENV-2 PDK53 (Supplementary Figs. 4c and 3b). In contrast, the TLR agonists induced strong immunological signatures involving immune cell trafficking, IFNs, and proinflammatory and T-cell responses.

In summary, although most features induced by viral infections were also induced by adjuvants, no single TLR agonist fully reproduced the viral signatures.

### Gene modules linking monocyte/Mφ, pro-inflammatory responses, and Th1 cytokines are associated with flavivirus infection

**Fig. 2:**
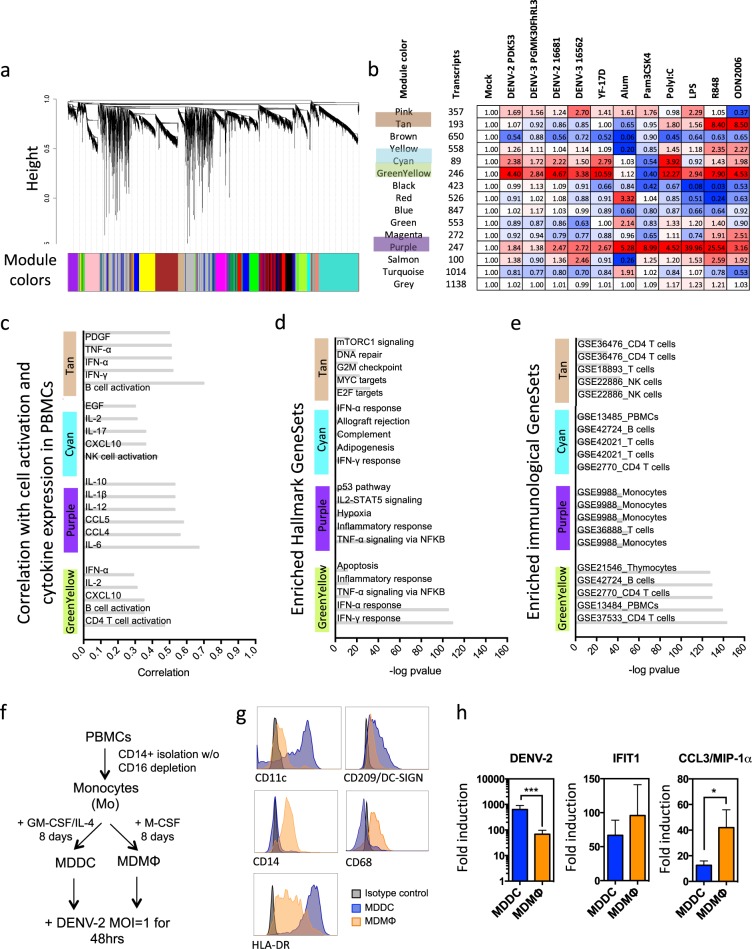
WGCNA analysis of gene modules induced by flavivirus infection identifies potential cell-specific signatures. **a**–**e** Gene expression data from all PBMCs infected with flaviviruses or treated with TLR agonists were used in WGCNA to identify gene modules induced by flavivirus infection. **a** Clustering dendrogram of genes with dissimilarity based on topological overlap used for module detection. Height represents the level of coexpression and color blocks indicate the gene modules identified. **b** Heatmap of module induction after flavivirus infection and TLR agonist treatment. Module induction was calculated as the average log_2_ expression of all genes in the module for all four donors after treatment normalized to the average log_2_ expression of all genes in the module for all four donors in mock-treated PBMCs. **c** Correlation of selected module expression with cytokine and cell activation. Data are shown here for modules tan, cyan, purple, and green–yellow. The full WGCNA correlation table is shown in Supplementary Fig. 5. **d**, **e** GSEA analysis of enriched hallmark genesets (**d**) and immunological genesets (**e**) in modules tan, cyan, purple, and green–yellow. **f**–**h** Cell-specific upregulation of CCL3/MIP-1α and IFIT1 transcripts after DENV-2 infection in monocyte-derived DCs (MDDCs) and macrophages (MDMΦs). **f** Experimental scheme. MDDCs and MDMΦs were generated from monocytes isolated from PBMCs of healthy human donors by culturing for 8 days in the presence in GM-CSF and IL-4 or M-CSF, respectively. **g** Confirmation of expression of CD11c, CD209/DC-SIGN, and HLA-DR on MDDCs and CD14 and CD68 and HLA-DR on MDMΦs immediately before infection. **h** Relative levels of DENV-2 RNA, IFIT1, and CCL3/MIP-1α transcripts at 48-h post infection with DENV-2 16681 at MOI = 1 measured by quantitative real-time RT-PCR and normalized to GAPDH. Data shown are the mean ± SEM of three independent donors. Significant differences are calculated using a two-tailed paired *t* test between MDDCs and MDMΦs. *p < 0.05, ***p < 0.005

To gain better insight into the potential links between the signatures identified, we performed an integrated analysis of the phenotypic, cytokine, and transcriptomic profiles using weighted correlation network analysis (WGCNA).^33^ The 48 microarray datasets from four donor PBMCs treated with viruses or adjuvants were used to construct a correlation network and identify gene modules with shared expression patterns, which were color-coded (Fig. 2a). We examined the average fold change expression of all genes in each module after treatment with viruses and TLR agonists (Fig. 2b). Module green–yellow showed the strongest activation by viruses, with an average fold change of gene expression >2 for all five viruses tested. Modules purple and cyan were also consistently induced. Importantly, these modules were not associated with pathogenicity as they were induced by both WT and LAV.

Expression of each module was related to external traits (activation of individual immune cell subsets and cytokine expression) and pathways analyzed using IPA and gene set enrichment analysis (GSEA)^34^ (summary in Fig. 2c–e, full table in Supplementary Fig. 5). Module green–yellow was strongly correlated with CD4 T-cell and B-cell activation, as well as with levels of the cytokines IFN-α, IL-2, and CXCL10/IP-10 (Fig. 2c). Transcripts in module green–yellow were categorized into IFN-α-induced and IFN-γ-induced genes under the control of STAT and IRF transcription factors in both GSEA and IPA analysis (Fig. 2d and Supplementary Fig. 6a–c). Module purple was associated with B-cell activation and levels of proinflammatory cytokines IL-6, IL-1β, TNF-α, and CCL3/4/5/11, as well as classical Th1 cytokines IL-12 and IFN-γ (Fig. 2c and Supplementary Fig. 5). Analysis of module purple transcripts revealed a significant enrichment in GSEA hallmark pathways such as TNF-α signaling through NF-κB and inflammatory responses (Fig. 2d), which were confirmed by IPA analysis (Supplementary Fig. 6d–f). Signatures in module cyan appeared to overlap with those of module green–yellow, with a strong component of IFN responses, even though it exhibited a unique negative correlation with NK-cell activation and expression of IL-17 and EGF (Fig. 2c–e). Module tan was also strongly associated with B-cell activation and levels of IFNs (Fig. 2c and Supplementary Fig. 5). However, at the transcript level, this module was not enriched in immune genes but mainly in genes involved in cell growth and metabolism (Fig. 2d). While modules green–yellow and purple were strongly induced after both virus and adjuvant treatments, module tan was upregulated by R848 and ODN2006 but not flavivirus infection (Fig. 2b), suggesting that distinct immune mechanisms might be involved in shaping B-cell activation.

One important observation from this analysis was that the inflammatory module purple triggered by flavivirus infection appeared to be mainly associated with monocytic cells, whereas other modules, such as IFN-related responses, were not assigned to a specific cell type when assessing the overlap with GSEA Immunological Genesets (Fig. 2e). To test this hypothesis experimentally, we generated human monocyte-derived DCs (MDDCs) and macrophages (MDMΦs), infected them with DENV-2 16881 and assayed after 48 h for induction of representative gene associated with NF-κB driven proinflammatory (CCL3/MIP-1α) and IFN signaling (IFIT1). While there was no difference in levels of IFIT1 between MDDCs and MDMΦs, CCL3/MIP-1α was upregulated at significantly higher levels in MDMΦs (Fig. 2f–h). Thus, MΦs are likely the major cell type producing the proinflammatory cytokine CCL3/MIP-1α while IFN responses are induced across DENV-infected cells.

### Specific immune signatures are associated with YF-17D immunogenicity

**Fig. 3:**
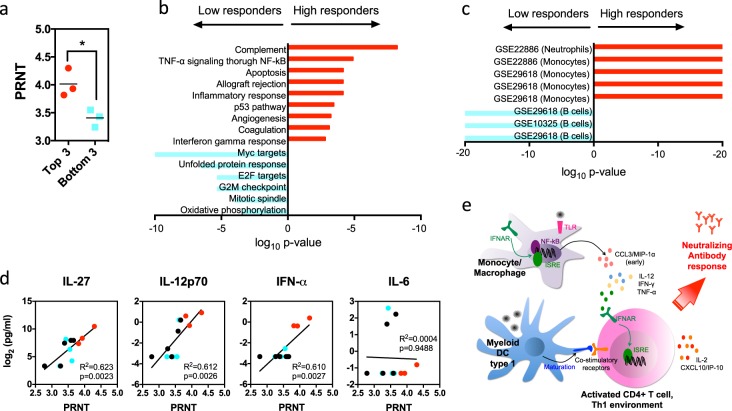
Signatures associated with nAb responses in YF-17D vaccinees. **a** nAb titers from the top three and bottom three responders for which microarray data were available in the cohort. Significant differences are determined using a two-tailed paired *t* test. *p < 0.05. **b**, **c** Top five pathways significantly enriched in comparing the transcriptomic signatures at 3 days post-YF-17D vaccination for three high responders and three low responders using GSEA Hallmark database (**b**) and GSEA Immunologic signature database (**c**). **d** Correlation plots between the log_2_ concentration of cytokines in the serum at 7 days post vaccination and the PRNT_50_ titer against YF-17D at 1-month post vaccination. One dot represents one individual. The three high responders and three low responders used in the GSEA analysis are highlighted in red and blue, respectively. The black dots represent the remaining individuals in the cohort whose responses were in between. A list of all cytokines analyzed is shown in Supplementary Fig. 7c. **e** Proposed model of immune signatures leading to high nAb responses to flavivirus infection

To evaluate which of these signatures could be associated with strong nAb responses after vaccination in humans, we used clinical data from a cohort of 11 flavivirus-naïve individuals immunized with YF-17D.^21^ PRNT_50_ titers in all individuals at one-month post vaccination, serum cytokine levels in all individuals at days 3 and 7 post vaccination, and whole blood microarray data for 7 of these 11 individuals at 3 days post vaccination were analyzed. Because the sample size was too small to apply WGCNA, we selected the top three responders versus bottom three responders (Fig. 3a) and performed GSEA analysis of the transcriptomic data to identify pathways differentially regulated between the two groups. Although innate immune signatures were induced in all individuals, consistent with our proinflammatory module predictions, the top responders showed significant enrichment of pathways associated with NF-κB signaling, inflammatory, and IFN-γ responses and monocyte gene signatures compared to the bottom responders (Fig. 3b, c). In contrast, the bottom responders showed enrichment of pathways linked to stress responses and cell proliferation with a strong B-cell component. PRNT_50_ was not correlated with viral load in this cohort (Supplementary Fig. 7a, b), suggesting that although high viral replication drives potent immune responses, it is not sufficient to explain the individual variations in nAb responses.

We also examined the levels of serum cytokines at 7 days post vaccination. Levels of IL-12, IFN-γ, TNF-α, CCL11/Eotaxin (associated with module purple in WGCNA), and IFN-α and IL-2 (associated with module green–yellow in WGCNA) were found to correlate with PRNT_50_ titers in the vaccinees (Fig. 3d and full table in Supplementary Fig. 7c). All these cytokines are generally linked to Th1 responses. None of the cytokines associated with other WGCNA modules correlated with nAb responses. Therefore, the gene signatures identified in the PBMC model can be largely validated following YF-17D vaccination in humans. Nonetheless, since other major cytokines associated with module purple such as IL-6, IL-10, and CCL5/RANTES did not correlate with the nAb response in vaccinees, it is possible that proinflammatory responses are involved indirectly through activation of T-cell responses.

In summary, the following early immune signatures after infection are associated with the generation of nAb responses in naïve individuals (Fig. 3e): (1) activation and maturation of mDCs, (2) a proinflammatory response driven by NF-κB transcription in monocyte/MΦs, (3) IFN signaling in multiple cell types, and (4) a Th1 cytokine environment.

### A combination of TLR1/2 and TLR3 agonists induces similar immune signatures as flavivirus

**Fig. 4:**
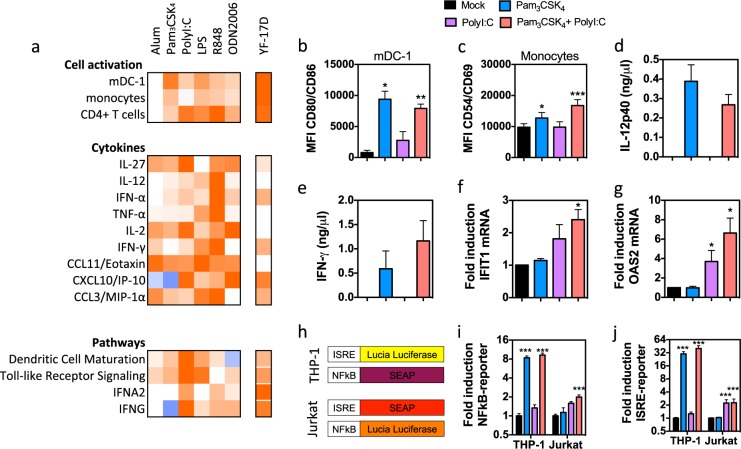
A combination of Pam_3_CSK_4_ and PolyI:C (PP) recapitulates signatures of high nAb responses. **a** Heatmap showing cell activation, cytokines, and pathway signatures associated with nAb responses induced in human PBMCs by alum and TLR agonists. The data were selected from the experiment presented in Supplementary Fig. 5 and compared to the same changes triggered by YF-17D in the same donor PBMCs, presented in Fig. 1. **b**–**g** Immune stimulation by individual and combination of Pam_3_CSK_4_ and PolyI:C in human PBMCs. **b**, **c** MFI of activation markers on mDC-1 and monocytes. Antibodies against activation markers were labeled with the same fluorophore. **d**, **e** Levels of IL-12 and IFN-γ in the supernatant. **f**, **g** Induction of IFN-stimulated IFIT1 and OAS2 mRNA by quantitative real-time RT-PCR, normalized to GAPDH. **h**–**j** Activation of reporter genes in reporter cell lines. **h** Schematic reporter constructs in THP-1 and Jurkat cell lines. **i**, **j** Induction of NF-κB and ISRE reporter in THP1 and Jurkat cells. Data shown are the mean ± SEM for four independent PBMC donors or two independent experiments in triplicate for cell lines. Significant differences are determined using a two-tailed paired *t* test between treatment and mock. *p < 0.05, **p < 0.01, ***p < 0.005

Next, we determined which TLR agonists could induce similar immune signatures in PBMCs based on the data in Supplementary Fig. 4. At the tested dose, Pam_3_CSK_4_ induced the strongest upregulation of activation markers on both mDC-1 and monocytes (Fig. 4a). PolyI:C potently activated the specific signatures of DC maturation, IFN-α and IFN-γ signaling, as well as IL-2 and CXCL10/IP-10 cytokines. Pam_3_CSK_4_, LPS, and R848 induced cytokines associated with the proinflammatory module. However, secretion of several of these cytokines in response to LPS and R848 was orders of magnitude higher than after YF-17D infection. In addition, R848 was linked in the WGCNA analysis to induction of modules tan, yellow, and magenta, which were not induced by flavivirus infection (Fig. 2b). Therefore, we selected the combination of Pam_3_CSK_4_ and PolyI:C (thereafter referred to as PP) as best reproducing the signatures of flavivirus infection.

We directly tested the PP combination in the PBMCs from four additional unrelated donors (Fig. 4b–g). PP induced similar or better responses than its individual adjuvants for all tested parameters: upregulation of CD80 and CD86 on mDC-1, CD54 and CD86 on monocytes (Fig. 4b, c), induction of Th1 cytokines IL-12 and IFN-γ (Fig. 4d, e), and upregulation of IFN-induced transcripts IFIT1 and OAS2 (Fig. 4f, g). We also tested the combination using NF-κB and IFN-stimulated response element (ISRE) reporters in monocytic THP-1 and T lymphocytic Jurkat cell lines (Fig. 4h). PP induced a strong NF-κB response in monocytes and ISRE response in both cell types (Fig. 4i, j). This confirms that the expected signatures were induced by PP.

### The PP adjuvant combination induces nAb responses in mice

**Fig. 5:**
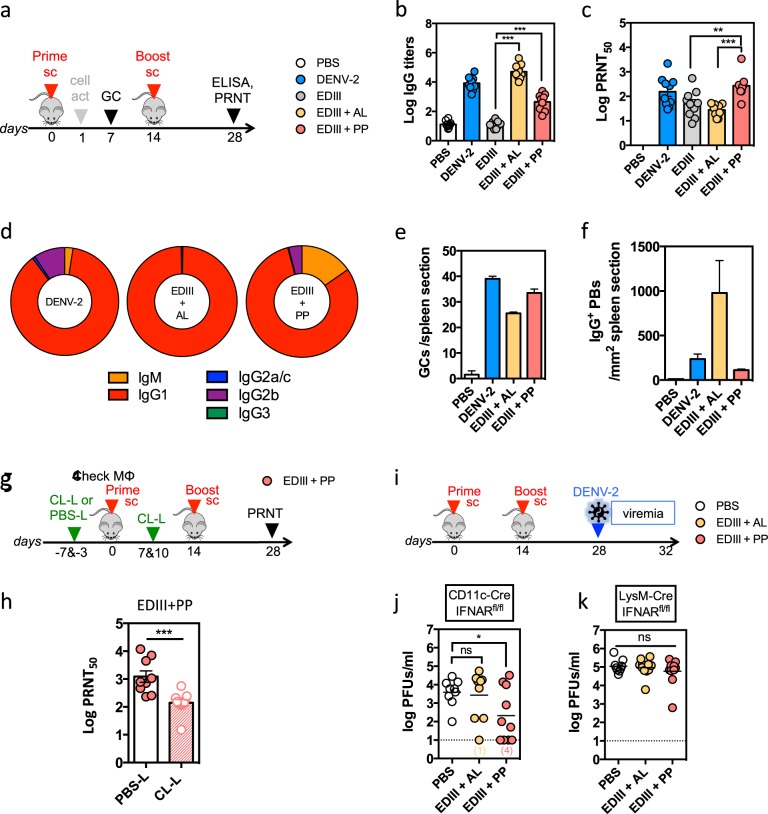
The PP adjuvant combination induces high nAb responses in mice, dependent on the presence of functional macrophages. **a**–**f** C57BL/6 mice were immunized twice at 14-day interval with DENV-2, EDIII alone or combined with AL or PP, and immune response analyzed as shown in **a**. **b** Log_10_ EDIII-specific IgG titers, (**c**) log_10_ PRNT_50_ titers, and (**d**) proportion of EDIII-specific IgM, IgG1, IgG2b, IgG2c, and IgG3 in the serum of the indicated mice at 28 days post vaccination (*n* = 9−10 mice per group from two independent experiments). Isotyping was done on pooled samples for each group. **e**, **f** Seven days post initial immunization, spleens were harvested from two mice per group and GCs detected by IFA. Representative images are shown in Supplementary Fig. 9. **e** Quantification of GCs in the entire spleen section for two mice per group. **f** Quantification of IgG^+^ cells (PBs) in two 1 mm^2^ areas for each spleen section. Data shown are the mean ± SEM. **g**–**h** C57BL/6 mice were treated with PBS-L or CL-L before immunization with EDIII + PP as shown in **g**. Cell depletion after CL-L treatment is shown in Supplementary Fig. 10. **h** Log_10_ PRNT_50_ titers at 28 days post vaccination (*n* = 9–10 mice per group from two independent experiments). **i**, **j** Conditional IFNAR knockout mice CD11c-Cre IFNAR^fl/fl^ and LysM-Cre IFNAR^fl/fl^ were immunized with EDIII + AL or EDIII + PP as shown in **i**. Viremia was analyzed by plaque assay in the serum 4 days after DENV-2 challenge in CD11c-Cre IFNAR^fl/fl^ (**i**) and in LysM-Cre IFNAR^fl/fl^ mice (**j**) (*n* = 8–10 mice per group from two independent experiments). Data shown are the mean ± SEM. Significant differences are determined by unpaired two-tailed *t* test. ns, not significant, *p < 0.05, **p < 0.01, ***p < 0.005

We determined whether the PP adjuvant combination could improve the immune response to vaccination using recombinant DENV-2 EDIII as a model dengue subunit vaccine. C57BL/6 mice were immunized subcutaneously twice at 14-day interval with a low dose of 5 µg of EDIII adjuvanted with PP or with alum plus MPLA (referred to as AL), a standard combination used in several commercialized vaccines (Fig. 5a).^25^ As control, C57BL/6 mice were infected subcutaneously with DENV-2 16681. Compared to AL, PP induced stronger activation of CD11b^+^ DCs (equivalent to mDC-1 in human), Ly6C^+^ monocytes (equivalent to classical human monocytes), and IFN-induced IFIT1 in the blood 1 day after vaccination (Supplementary Fig. 8).

The antibody response was quantified 14 days after the boost. When total EDIII-specific IgG was measured by ELISA, EDIII without adjuvant did not induce any significant response. The addition of AL or PP both increased IgG titers, although the response to AL was two orders higher than PP (Fig. 5b). DENV-2 also induced a high level of antigen-specific IgG response. In contrast, when nAbs were measured by PRNT, addition of PP induced higher PRNT_50_ titers than AL, reaching levels similar to those induced by immunization with DENV-2 (Fig. 5c). The addition of AL induced predominantly an IgG1 response to EDIII, whereas the addition of PP also induced significant proportions of IgM and IgG2b leading to an isotype profile more similar to the response to DENV-2 (Fig. 5d). We analyzed germinal center (GC) and IgG^+^ PB formation in the spleen 7 days after the first immunization. DENV-2, EDIII + AL, and EDIII + PP induced comparable numbers of GCs, although the GCs were smaller in size in mice immunized with EDIII + PP (Fig. 5e and Supplementary Fig. 9). However, EDIII + AL induced more IgG^+^ PBs compared to EDIII + PP or DENV-2 immunization (Fig. 5f). These results suggest that PP induces a superior nAb responses to EDIII than AL, reaching a similar nAb level as induced by virus infection.

To investigate the requirement of MΦs in nAb responses to PP, we used clodronate liposomes (CL-L) to deplete MΦs in C57BL/6 mice prior to immunization (Fig. 5g).^35^ Injection of CL-L before EDIII + PP, and then again before the boost, specifically reduced the numbers of F4/80^+^ and CD169^+^ MΦs without affecting CD11c^+^ DCs or CD19^+^ B cells in the spleen (Supplementary Fig. 10). Resulting nAb titers were approximately tenfold lower in CL-L-treated mice than mice treated with control PBS-L (Fig. 5h), indicating that MΦs are required for generating nAb responses to EDIII + PP vaccination.

We further examined the requirement for MΦ-specific innate immune signaling for the induction of protective responses in a challenge model. We used the recently described CD11c-Cre^+/−^ IFNAR^fl/fl^ and LysM-Cre^+/−^ IFNAR^fl/fl^ mice, which lack IFN-α receptor exclusively in CD11c^+^ DCs and LysM^+^ MΦs, respectively. These mice support productive DENV-2 infection and remain capable of mounting antibody and T-cell responses to subunit vaccines.^36^ Both strains of mice were immunized subcutaneously at days 0 and 14 with EDIII + AL or EDIII + PP (5 μg EDIII per dose) and challenged intraperitoneally on day 28 with DENV-2 D2Y98P (10^6^ PFUs). Viremia was analyzed 4 days later (Fig. 5i). In CD11c-Cre^+/−^ IFNAR^fl/fl^ mice, immunization with EDIII + AL did not significantly reduce DENV-2 viremia compared to unvaccinated controls, whereas EDIII + PP did confer a significant, albeit partial, protection against DENV-2 challenge (Fig. 5j). In LysM-Cre^+/−^ IFNAR^fl/fl^ mice, immunization with EDIII + AL or EDIII + PP did not reduce DENV-2 viremia when compared to unvaccinated mice (Fig. 5k), indicating that IFN responses in MΦs are required for nAb responses. Taken together, the results from different mouse models show the superiority of PP as adjuvant in inducing nAb response and the requirement for MΦ in responses to EDIII + PP vaccination.

### PP elicits more consistent nAb and T-cell responses in nonhuman primates (NHPs), but only confers partial protection against virus challenge

**Fig. 6:**
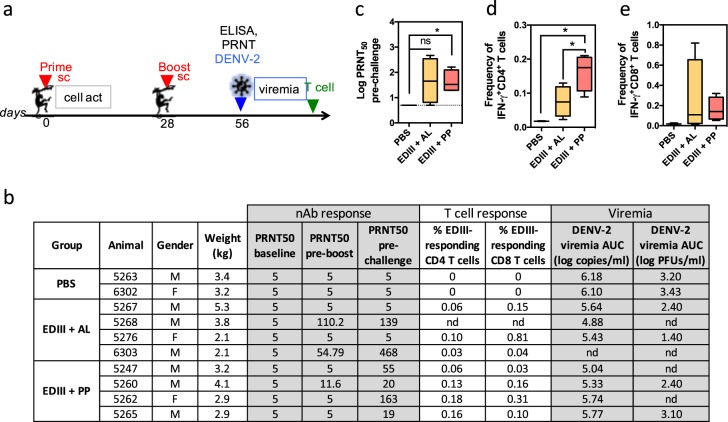
The PP adjuvant combination elicits more consistent nAb and T-cell responses in NHPs. **a** Immunization and challenge scheme in cynomolgus macaques. **b** Responses of individual NHPs to vaccination and challenge. Columns 1–4: animal identification number, sex (M/F), and body weight. Columns 5–7: reciprocal DENV-2 PRNT_50_ titer in the serum at day 1 (pre vaccination), day 28 (pre boost), and day 56 (pre challenge). PRNT_50_ below detection value are noted as 5. Columns 8 and 9: frequency of EDIII-specific IFN-γ responsive CD4^+^ and CD8^+^ T cells in the spleen at day 63. Columns 10 and11: Viremia, defined as the area under the curve (AUC) of DENV-2 titers in the serum of challenged animals between days 56 and 53 (days 1–7 post challenge) measured by qRT-PCR and plaque assay. **c** Log_10_ DENV-2 PRNT_50_ titer by group at day 56 post vaccination. **d**, **e** Frequency of EDIII-specific IFN-γ responsive CD4^+^ (**d**) and CD8^+^ (**e**) T cell by group at day 63. Data shown are box plots depicting the median, 25th and 75th percentiles with whiskers showing the min to max distribution for each group. Significant differences are determined by unpaired two-tailed *t* test. ns: not significant, *p < 0.05

Next we vaccinated and challenged cynomolgus macaques. Ten DENV-2-naïve animals were divided into three groups: two were given PBS (control), four were immunized subcutaneously with EDIII + AL, and four with EDIII + PP twice at 28-day interval (Fig. 6a). Because PolyI:C is not stable in the circulation of NHPs and humans, PolyICLC was used in the PP formulation.^37^ Both vaccines were well tolerated, with no clinical manifestations more severe than a mild reaction at injection site, no fever, and no elevation in alanine transferase (ALT) levels between days 1 and 7 post vaccination (Supplementary Fig. 11a, b). Levels of serum proinflammatory cytokines CCL2/MCP-1 and CXCL10/IP-10, induction of IFIT1 in the blood and expression of CD80 and CD86 by mDCs were significantly higher in EDIII + PP compared to EDIII + AL immunized NHPs at day 1 post vaccination (Supplementary Fig. 11c–f). However, serum levels of Th1 cytokines IFN-γ and TNF-α were only upregulated in two of four EDIII + PP immunized NHP, and no upregulation of activation marker CD86 was detected on blood monocytes after vaccination (Supplementary Fig. 11g–i). These results indicate that, at the dose used, PP induced some but not all expected immune signatures in NHPs. We also noted that one animal (6303) in the EDIII + AL group showed high levels of serum IFN-γ and TNF-α before and throughout day 7 post immunization (Supplementary Fig. 11g, h), indicating a specific immune status potentially due to an unrelated concomitant infection.

EDIII-specific IgG was analyzed by ELISA and nAb titers by PRNT over time. After priming, the AL formulation induced significantly higher IgG titers than PP; however, unlike in mice there was no difference between the two groups after the boost **(**Supplementary Fig. 12a, b). Nonetheless, as in mice the ratio of EDIII-specific IgG1 versus total IgG was higher in the AL than PP vaccinated NHPs (Supplementary Fig. 12c). One month after the boost, two animals vaccinated with EDIII + AL had high nAb titers, while the other two did not have detectable nAbs (Fig. 6b). In the EDIII + PP immunized group, all animals developed nAb titers. Overall, only the EDIII + PP group had a significant nAb response after two immunizations (Fig. 6c). In all vaccinated animals, nAb titers did not correlate to the total IgG or IgG1 titers (Supplementary Fig. 12d, e).

At day 56, NHPs were challenged subcutaneously with 10^6^ PFUs of DENV-2 16681. Viremia was measured by plaque assay and viral RNA quantified by real-time PCR in the serum daily for 7 days. In the control animals, both viremia and viral RNA were detected for 6 or 7 days following challenge (Fig. 6b and Supplementary Table 1). Both EDIII + PP and EDII + AL vaccinated animals had a reduced level of viremia, indicating partial protection against virus challenge; however, PP did not perform better than AL. The only animal that was completely protected (no virus detected by either method) was from the EDIII + AL group (6303). This was the animal with the highest nAb titers but also high baseline levels of Th1 cytokines. Thus, the specific immune status of this animal might have influenced nAb responses and protection independently of the EDIII + AL vaccination.

To assay EDIII-specific T-cell response, splenocytes were analyzed at 7 days post challenge for IFN-γ and TNF-α production by CD4^+^ and CD8^+^ T cells in response to antigen stimulation. Only one animal immunized with EDIII + AL had a consistent CD4^+^ T-cell response, characterized by an increased frequency of CD4^+^ T cells producing IFN-γ or IFN-γ plus TNF-α upon stimulation with EDIII and live DENV-2 (Supplementary Fig. 13). On the other hand, three of four animals vaccinated with EDIII + PP had such a CD4^+^ T-cell response. Overall, the antigen-specific CD4^+^ T-cell response was significantly superior in response to PP than AL adjuvants (Fig. 6d). A similar trend was observed for the CD8^+^ T-cell response, even though the strongest responder was from the EDIII + AL vaccinated group (Fig. 6e and Supplementary Fig. 13d–f). Taken together, these results show that the PP adjuvant combination elicits more consistent nAb, CD4^+^ and CD8^+^ T-cell responses to EDIII vaccination than AL, but that in our experimental setting these levels of nAb and T-cell responses are still insufficient to protect against viral challenge.

## Discussion

In this study, we (1) determined immunological signatures that are induced by flavivirus infection using a PBMC model system, (2) identified a TLR agonist combination that mimics the same signatures, and (3) tested the ability of this combination to stimulate nAb responses to a model dengue subunit vaccine and protection against virus challenge in both mice and NHPs.

We developed a simple system to study the early immune signatures to flaviviruses in humans using infection of PBMCs in vitro. The PBMC system showed a strong overlap with the gene expression and cellular changes induced in the blood of human subjects following YF-17D vaccination. The PBMC system made it possible to study a panel of TLR agonists and flaviviruses known to induce nAbs in humans using a series of assays, including upregulation of activation markers, cytokine secretion, and transcriptional profiling. Our studies identified signaling through IFNs, proinflammatory cytokines, TLRs, and acute phase response, and DC maturation as common immune signatures induced by flavivirus infection. TLR agonists also induced most signatures but no single TLR agonist was able to induce the full spectrum of signatures.

Our integrated bioinformatic analysis of the phenotypic, cytokine, and transcriptomic profiles from PBMCs and a cohort of flavivirus-naïve individuals immunized with YF-17D identified immune signatures that are associated with nAb responses. The combination of TLR2 agonist Pam_3_CSK_4_ and TLR3 agonist PolyI:C (PP) best reproduced these signatures. Some identified signatures were expected, such as DC activation and maturation and IFN responses, others were unexpected, such as a positive association with monocyte/Mφ-driven pro-inflammatory responses and Th1 cytokines, or a negative association of nAbs with B-cell responses. Nonetheless, these results are consistent with previous reports showing that the frequency of circulating PBs is associated with total IgG, but not nAb titers in acute dengue patients.^16,18^ The importance of a Th1 environment has also been suggested. In mice, higher IFN-γ and IgG2b responses are associated with induction of YF-17D nAbs.^38^ In humans, high frequency of virus-specific CD4^+^ IFN-γ^+^ T cells at early time points is associated with a strong nAb response to YF-17D vaccination.^39^ Levels of IFN-γ, IL-12, and IFN-α are also found to be higher in dengue patients with mild fever compared to those with more severe disease,^40–42^ indicating that a Th1 cytokine environment might also be related to protection. Together, this suggests that induction of high nAb titers after flavivirus infection might depend on immune mechanisms that differ from the classical model of B-cell maturation and expansion leading to high antigen-specific IgG titers.

Although monocytes/Mφ have received considerable attention in flavivirus infection due to their role in antibody-dependent enhancement of infection, their role in driving immune responses has not been thoroughly examined. We showed that in vitro, human MΦs are likely the major cell type producing proinflammatory cytokines. MΦs were also critical in the generation of immune responses after immunization with a PP-adjuvanted formulation. In mice, depletion of macrophages by chlodronate liposomes before EDIII + PP immunization reduced nAb titers by approximately tenfold. Furthermore, EDIII + PP immunization conferred partial protection in CD11c-Cre^+/−^ IFNAR^fl/fl^ mice, which lack IFN-α receptor exclusively in CD11c^+^ DCs, but not in LysM-Cre^+/^^−^ IFNAR^fl/fl^ mice, which lack IFN-α receptor in LysM^+^ MΦs. This was consistent with previous reports of less effective protective responses against DENV in the LysM-Cre^+/−^ IFNAR^fl/fl^ model, and associated with lower IFN-γ levels.^36^ Differences in immunostimulatory capacity of DCs and Mφs were also observed after *Leishmania* infection, with Mφs being more effective than DCs or B cells in eliciting Th1 responses.^43^

How could Mφs and a Th1 cytokine environment be more conducive to nAb responses? It is possible that endogenous cellular differences and/or modification of the cellular environment by cytokines differentially affect antigen trafficking, processing, and presentation. Differences in antigen-presenting cells could also play a role, as subcapsular sinus Mφs are known to present intact antigens to follicular B cells, whereas DCs generally present processed antigens.^17^ It is also possible that the cellular and cytokine environment induces differential antibody class switching or glycosylation, influencing how efficiently the virus is neutralized even through binding at the same epitope. Finally, the Th1 environment could preserve better the diversity of Ab response by limiting clonal selection and PB differentiation, which might lead to the overproduction of a small number of highly reactive, but poorly neutralizing Ab clones.

To determine whether reproducing flavivirus immune signatures with the PP adjuvant combination could be useful for DENV vaccine development, we tested the ability of PP to stimulate nAb responses using the recombinant DENV-2 EDIII domain as a test subunit antigen. For comparison, we used alum and TLR4 agonist MPLA (AL), a standard adjuvant used in many vaccines. In mice, significantly higher level of nAbs and higher protection against DENV-2 challenge were induced by EDIII + PP immunization than EDIII + AL vaccination, in spite of a lower level of overall antigen-specific IgG. In NHPs, EDIII + PP but not EDIII + AL consistently induced both nAb and T-cell responses in all animals. These results clearly show that mimicking immune signatures associated with nAb responses with TLR agonists (PP) has the potential to stimulate broader protective immune responses.

The levels of protection upon challenge, however, remained intermediate. Compared to nonimmunized mice or NHPs, EDIII + PP, but not EDIII + AL, vaccination induced significant protection against DENV-2 challenge. However, no significant difference in protection was observed between EDIII + PP and EDIII + AL vaccinations, raising concerns on the limitations of the current studies, especially translation to humans. On one hand, the lack of significant difference in protection between EDIII + PP and EDIII + AL vaccinations could be due to the choice of AL as adjuvant control, because after all AL is one of the widely used adjuvants for vaccination in humans because of its potency. On the other hand, the experimental methodology and design of the current studies were not optimal to induce maximal immune responses so as to confer better protection. In mice, the partial protection could be due to the low dose of antigen (5 μg) and the two immunizations, versus typically three or four injections of at least 50 μg of antigens in most previous studies. In macaques, not all animals reached the nAb threshold needed for protection (an apparent >1:50 in our cynomolgus macaque DENV-2 infection model) and only limited numbers of animals were used in each arm of the experiment due to the cost and limited availability of the NHPs. In humans, the correlation between nAb induction and specific immune signatures in the current study was derived from a very small number of YF-17D vaccinated individuals, which could have overestimated the importance of the correlation. This could be significant considering that other immune signatures, besides the strong monocyte/MΦ-driven responses and Th1 cytokine environment, were also correlated with nAb induction, although weakly. In addition, EDIII does not contain important quaternary neutralizing epitopes or potent T-cell epitopes that may be needed for protective immunity in NHPs and humans. Despite these caveats, the more consistent and broad nAb and CD4^+^ T-cell responses induced by EDIII + PP highlights the potential of combining our approach with antigen optimization in dengue and other vaccine development.

## Materials and methods

### Ethics statement

Research involving human PBMCs was approved by the Institutional Review Board (NUS IRB 10-285). The YF-17D trial was previously published.^21^ Trial approval was obtained from Singhealth Centralized Institutional Review Board (ID:2013/385/E) and is registered under clinicaltrials.gov registration no. NCT01943305. Animal experiments performed at NUS (C57BL/6 mice), A*STAR (IFNAR mice) and SingHealth (NHP) were approved by the respective Institutional Animal Care and Use Committee and conducted following institutional guidelines.

### Viruses and adjuvants

DENV-2 16681 (U87411.1), DENV-2 PDK53 (M84728.1), DENV-3 16562, DENV-3 PGMK30FhL3,^28^ and YF-17D (X03700.1) were produced in Vero (African green monkey) cells for the systems biology study. DENV-2 16681 and D2Y98P (JF327392.1) were produced in C6-36 (Aedes albopictus) cells for challenge experiments. Adjuvants were obtained from Invivogen (Vaccigrade) and used at the following concentration: Alhydrogel^®^ 2%, Pam_3_CSK_4_ 2.5 μg/ml, PolyI:C 5 μg/ml, LPS 1 μg/ml, R848 2.5 μg/ml, and ODN2006 5 μg/ml.

### Systems biology study

PBMCs from four healthy, dengue-naïve donors (two male and two female donors, aged 18–40 years old) were isolated by Ficoll density gradient. A total of 10^7^ cells per condition were treated with viruses at MOI = 1 and adjuvants at the above concentrations. After 48 h, cells were used for analysis by flow cytometry and microarray and supernatant collected for cytokine profiling.

### Flow cytometry

PBMCs were diluted in FACS buffer (PBS, 0.5% BSA, 0.05% sodium azide) at 10^6^ cells/100 μl and stained for 1 h at 4 °C with antibody cocktails as described in Supplementary Table 2. Cells were washed twice and nuclei counterstained with DAPI before acquisition on a BD LSRII cytometer. Results were analyzed with FlowJo.

### Microarray analysis

Total RNA was extracted from 106 PBMCs with Trizol (Ambion). cRNA libraries were generated using the Illumina TotalPrep RNA Amplification Kit (Ambion) and hybridized to an Illumina HumanRef-12 V4 BeadChip (Illumina) at 55 °C for 18 h, according to the manufacturer’s instructions. After washing, blocking, and staining with streptavidin-Cy3, a high-resolution Illumina Bead Array Reader confocal scanner (Illumina) was used to scan the chip. Genome Studio (Illumina) was used to subtract background from raw gene expression intensity data. Standard normalization procedures for one-color array data and data analysis to identify differentially expressed genes were performed using the GeneSpring GX software, version 12.5 (Agilent Technologies). Significantly regulated genes (*p* < 0.05 with a cutoff of 1.5-fold change in treated compared to untreated samples) were analyzed with IPA^44^ and GSEA.^34^

### Cytokine profiling

Fluorescent bead measurement of cytokines, chemokines, and growth factors in PBMC supernatents was performed using the Luminex technology xMAP (Bioplex 27-plex human cytokine kit, Bio-Rad) as per the manufacturer’s instructions. The standard curves were optimized automatically by the software (Bioplex manager) and verified manually. In order to prevent batch effect, samples were randomized prior to analysis. Calibrations and validations were performed prior to analyses. In experiments performed to validate the activity of combinations of adjuvants, measurement of cytokine levels in human PBMC supernatants (IL-12p40, IFN-γ) was done by ELISA (Biolegend) following the manufacturer’s recommendations.

### WGCNA analysis

The recommended basic data processing and analysis procedure was applied to our datasets.^33^ Data from 48 microarrays (mock, virus-infected, and adjuvant-treated from four individual donors) expressed as log_2_ fold change for each of 47,323 probes were processed by removing undetected probes and selecting probes with the 70% highest variance. Sample clustering detected no outliers. Module identification was performed using a signed network with power = 9, mergeCutHeight = 0.2, and minModuleSize = 30. Module eigengenes were correlated by Pearson correlation with external traits. The following data were used as weights for external traits: MFI of activation markers for immune cell activation and concentration in pg/ml in the supernatant for cytokines. The genes in relevant modules were analyzed by IPA and GSEA.

### Infections of MDDCs and MDMΦs

Monocytes were isolated from PBMCs using the EasySepTM human monocyte enrichment kit without CD16 depletion (StemCell) and differentiated into MDDCs in the presence of 25 ng/ml GM-CSF + 50 ng/ml IL-4 (R&D Systems) or MDMΦs with 25 ng/ml M-CSF (R&D Systems) for 8–10 days. The phenotype of differentiated cells was confirmed by flow cytometry using the panel of antibodies in Supplementary Table 3 (MDDCs: CD14- CD68- HLADR++ CD11c+ CD209/DC-SIGN+ and MDMΦs: CD14+ CD68+ HLADR+ CD11c- CD209/DC-SIGN-) before infection with DENV-2 16681 at MOI = 1 for 48 h.

### NF-κB and ISRE reporter cell lines

THP-1 and Jurkat-Dual reporter cell lines were purchased from Invivogen and handled following the manufacturer’s recommendation.

### DENV-2 EDIII expression

DENV-2 PDK53 residues 296–396 were used to express recombinant DENV-2 EDIII. Briefly, the EDIII sequence was amplified from cDNA obtained from virus stocks using primers 5′ CACCAGTTACTCAATGTGTACCGGCAAG 3′ and 5′ CTATGACCCCTTCTTAAACCAGTTCAG 3′, then cloned in pET-TOPO-151 (Invitrogen) to add a N-terminal 6xHis tag and a TEV protease recognition site. The plasmid was transformed in E.Coli BL21(DE3) and expression induced with 0.25 mM IPTG. After 16-h incubation at room temperature, bacteria were lysed with BugBuster mastermix (Millipore) and centrifuged at 11,000 *g* for 30 min. Pellet was resuspended in lysis buffer supplemented with 8 M urea, bound to HisPur resin (ThermoFisher) and recombinant protein eluted using a 50 m M–1M gradient imidazole in wash buffer (30 mM sodium phosphate, 300 mM NaCl, 10% glycerol, and 8 M urea). Fractions containing EDIII as determined by SDS-PAGE and Coomasie blue staining were dialized against storage buffer (30 mM sodium phosphate, 300 mM NaCl, and 10% glycerol). Folding was verified by ELISA using the 3H5 antibody (Millipore). For NHP immunizations, the 6xHis tag was removed using the TEV protease (Invitrogen) following the manufacturer’s recommendations.

### Animal experiments

#### Mice

C57BL/6 mice were obtained from InVivos (Singapore). CD11c-Cre IFNAR1^fl/fl^ mice and LysM-Cre IFNAR1^fl/fl^^36^ were bred at Agency of Science, Technology and Research (A*Star, Singapore). Mice (M/F, 7–10 weeks old) were immunized s.c. with 10^6^ PFUs DENV-2 16681, 5 μg DENV-2 EDIII + 20 μl Alhydrogel^®^ 2% + 10 μg MPLA (EDIII + AL) or 5 μg DENV-2 EDIII + 10 μg Pam_3_CSK_4_ + 50 μg PolyI:C (EDIII + PP) following the indicated schedules. Challenge was performed by i.p. injection of 10^6^ PFUs D2Y98P. For depletion of MΦs, C57BL/6 mice were injected twice with 100 μl clodronate (CL-L) or control (PBS-L) liposomes (http://www.clodronateliposomes.org), s.c. 7 days before and i.p. 3 days before immunization. Innate immune responses to the adjuvanted vaccines were analyzed in whole blood by flow cytometry and quantitative real-time RT-PCR, antibody responses by ELISA and PRNT, GC formation by immunofluorescence of spleen cryosections, and DENV-2 viremia after challenge by quantitative real-time PCR and plaque assay as described below.

#### Nonhuman primates

Locally caught cynomolgus macaques (M/F, 3–5 kg) were housed at the SingHealth Experimental Medicine Center. Animals were immunized s.c. with 50 μg DENV-2 EDIII + 200 μl Alhydrogel^®^ 2% + 100 μg MPLA (EDIII + AL) or 50 μg DENV-2 EDIII + 100 μg Pam_3_CSK_4_ + 500 μg PolyICLC (EDIII + PP). PolyICLC was prepared as previously described.^37^ Challenge was performed by s.c. injection of 10^6^ PFUs DENV-2 16681. The response to vaccination in NHP was followed during 7 days after the first immunization. Rectal temperature was measured and animals were observed daily for food and water intake, reaction at site, and systemic adverse events. ALT and cytokine levels were measured in the serum using an ALT enzymatic kit (Abcam) and the LegendPlex NHP chemokine/cytokine 13-plex (Biolegend) following the manufacturer’s recommendations. Innate immune responses to the adjuvanted vaccines were analyzed in whole blood by flow cytometry and quantitative real-time RT-PCR antibody responses by ELISA and PRNT, DENV-2 viremia after challenge by quantitative real-time PCR, and plaque assay and T-cell responses by intracellular staining of antigen-stimulated splenocytes as described below.

#### Analysis of cell activation by flow cytometry in whole blood

Whole blood was subjected to RBC lysis using ACK buffer. Cells were diluted in FACS buffer (PBS, 0.5% BSA, 0.05% sodium azide) at 10^6^ cells/100 μl and stained for 1 h at 4 °C with antibody cocktails as described in Supplementary Table 4. Cells were washed twice and nuclei counterstained with DAPI before acquisition on a BD LSRII cytometer. Results were analyzed with FlowJo.

#### Analysis of gene expression in whole blood

Whole blood was subjected to RBC lysis using ACK buffer and cells lysed in RLT buffer (Qiagen). Quantification of host transcripts was performed by as previously described.^45^ Results are expressed as the fold change normalized to GAPDH and to untreated sample using the 2^−ddCt^ method. The list of primers used is shown in Supplementary Table 5.

#### ELISA

ELISA plates (Nunc) were coated with 2 μg/ml DENV-2 EDIII in PBS, overnight at 4 °C. The next day, plates were washed once and blocked in 5% nonfat milk for 1 h, then incubated with serial dilutions of heat-inactivated serum in 1% nonfat milk for 2 h at 37 °C. Depending on the experiment, the following secondary antibody coupled to HRP were incubated for 1 h at room temperature: goat polyclonal anti-mouse total IgG (Biolegend), mouse anti-human/monkey IgG (clone G18-145, BD), goat polyclonal anti-mouse IgM, IgG1, IgG2b, IgG3 (Abcam), or IgG2c (Pierce), and mouse anti-human/monkey IgG1 (clone HP6069, Thermo). TMB substrate was added for 5 min and reactions stopped with 2 N H2SO4. OD450 values were fitted with a nonlinear regression with variable slope (four parameters) in GraphPad Prism and antibody titers were defined as the lowest dilution with signal two times above background. Statistical differences are evaluated based on the log_10_ antibody titers.

#### PRNT

Plaque reduction neutralization assay was performed as previously described.^21^ Briefly, BHK-21 cells were maintained in RPMI + 10% FBS and seeded in 24-well plates for the assay. Serial dilutions of heat-inactivated serum were pre incubated with 50 PFUs of DENV-2 16681 for 2 h at 37 °C before allowing infection of the cell monolayer for 1 h. Cells were overlayed with maintenance media supplemented with 1% CMC and plaques stained 5 days later with 1% crystal violet. Curves were fitted in GraphPad Prism using a four parameter logistic regression and the PRNT_50_ calculated as the serum concentration leading to a 50% reduction in plaques from the virus only control. Statistical differences are evaluated based on the log_10_ PRNT_50_.

#### Quantification of DENV-2 viremia

The quantification of DENV-2 viral load in the serum of challenged animals was performed following standard protocols previously published. Briefly, plaque assay was performed by infecting monolayers of BHK-21 cells with serial dilutions of serum, overlaying with maintenance media supplemented with 1% CMC, and staining plaques 5 days later with 1% crystal violet. Quantitative real-time RT-PCR was performed following the CDC assay.^46^ For both methods, the area under the curve and the peak value of titer for the first 7 days after challenge were calculated using GraphPad Prism.

#### Immunofluorescence of mouse spleen cryosections

Mouse spleens were collected at 7 days post immunization, embedded in OCT medium and frozen at −80 °C before processing into 5 μm sections. Samples were fixed in cold 4% paraformaldehyde for 10 min followed by blocking in 5% FBS for 1 h at room temperature. For GC analysis, Alexa-Fluor 594 B220 (Biolegend 103254), Alexa-Fluor 488 anti-mouse IgG (Biolegend 405319), and Alexa-Fluor 647 anti-GL-7 (Biolegend 144605) or for macrophage depletion, Alexa-Fluor 594 B220, FITC anti-mouse CD68 (Biolegend 137005) /FITC anti-mouse CD11c (Biolegend 117305), and Alexa-Fluor 647 anti-mouse CD169 (Biolegend 142407) were incubated at 1:100 overnight at 4 °C. Samples were mounted on glass slides in ProLong Antifade reagent (Invitrogen). Images were acquired on a Zeiss LSM700 confocal microscope and on a Mirax Midi slide scanner system for whole organ imaging. GCs were defined as discrete areas of B220, GL-7, and IgG signal colocalization and counted manually in each spleen section. PBs were defined as IgG^+^ cells and quantified in two 1 mm^2^ areas in each spleen section using ImageJ.

#### T-cell intracellular staining of NHP splenocytes

Splenocytes from NHPs were processed on ice immediately after killing. A total of 10^6^ cells per condition were left unstimulated or restimulated for 16 h with 10 μg/ml EDIII, 10^7^ PFUs DENV-2 16681, or 20 ng/ml PMA + 1ug/ml ionomycin as a positive control. Brefeldin A and monensin (Biolegend) were added at 1:2000 each for the last 5 h of stimulation. Cells were incubated with Near IR Live/Dead stain (Thermo), fixed with Cytoperm/Cytofix (BD), and then stained with CD3, CD4, CD8, IFN-γ, and TNF-α antibodies as described in Supplementary Table 6. Cells were acquired on the Attune NxT flow cytometer (Thermo) and results analyzed with FlowJo. The T-cell response was considered significant if the frequency of responding cells was greater than threefold that of the average of unstimulated cells.

### Statistical analysis

The method used for statistical analysis is indicated in each figure legend. More detailed description of the statistical methods used in microarray and WGCNA analysis are available in the respective methods section.

### Reporting summary

Further information on experimental design is available in the Nature Research Reporting Summary linked to this article.

## Supplementary information

Supplementary Materials

Reporting Summary

## Data Availability

Microarray data were deposited in NCBI GEO under the accession number GSE131818.

## Supplementary information

**Supplementary Information** accompanies the paper on the *npj Vaccines* website (10.1038/s41541-019-0119-3).
