# Author Correction: Mimicking immune signatures of flavivirus infection with targeted adjuvants improves dengue subunit vaccine immunogenicity

**DOI:** 10.1038/s41541-019-0140-6

**Published:** 2019-10-24

**Authors:** Katell Bidet, Victor Ho, Collins Wenhan Chu, Ahmad Nazri Mohamed Naim, Khaing Thazin, Kuan Rong Chan, Jenny G. H. Low, Milly M. Choy, Lan Hiong Wong, Paola Florez de Sessions, Yie Hou Lee, Martin L. Hibberd, Eng Eong Ooi, Katja Fink, Jianzhu Chen

**Affiliations:** 1 Interdisciplinary Research Group in Infectious Diseases, Singapore-MIT Alliance for Research and Technology, Singapore, Singapore; 2 Singapore Immunology Network, Agency for Science, Technology and Research, Singapore, Singapore; 3 School of Biological Sciences, Nanyang Technological University, Singapore, Singapore; 4 Genome Institute of Singapore, Agency for Science, Technology and Research, Singapore, Singapore; 5 Department of Biological Sciences, National University of Singapore, Singapore, Singapore; 6 Emerging Infectious Diseases, Duke-NUS Graduate Medical School, Singapore, Singapore; 7 Department of Infectious Diseases, Singapore General Hospital, Singapore, Singapore; 8 KK Women’s and Children’s Hospital, Singapore, Singapore; 9 Department of Pathogen Molecular Biology, London School of Hygiene and Tropical Medicine, London, WC1E 7HT UK; 10 Koch Institute for Integrative Cancer Research and Department of Biology, Massachusetts Institute of Technology, Cambridge, MA 02139 USA

**Keywords:** Adjuvants, Immunology

**Correction to:** *npj Vaccines* 10.1038/s41541-019-0119-3, published online 25 June 2019

The original version of this Article contained an error in the spelling of the author Ahmad Nazri Mohamed Naim, which was incorrectly given as Ahmad Nazri Hohamed Naim. This has now been corrected in both the PDF and HTML versions of the Article.

