# Supplementary material for: Mimicking immune signatures of flavivirus infection with targeted adjuvants improves dengue subunit vaccine immunogenicity
Source: NPJ Vaccines. 2019 Jun 25;4:27. doi: 10.1038/s41541-019-0119-3 (PMC6592935; doi:10.1038/s41541-019-0119-3)
Supplement: Supplementary file 1 — Supplementary Materials [file 41541_2019_119_MOESM1_ESM.pdf]

Supplementary materials

Mimicking immune signatures of flavivirus infection with targeted adjuvants improves dengue subunit vaccine immunogenicity in mice and primates

Katell Bidet[1], Victor Ho[2,3], Collins Wenhan Chu[4], Ahmad Nazri Mohamed Naim[4], Khaing Thazin[5], Kuan Rong Chan[6], Jenny G.H. Low[6,7], Milly M. Choy[4], Lan Hiong Wong[1], Paola Florez de Sessions[4], Yie Hou Lee[8], Martin L. Hibberd[9], Eng Eong Ooi[1,6], Katja Fink[2,3], Jianzhu Chen[1,10*].

[1] Interdisciplinary Research Group in Infectious Diseases, Singapore-MIT Alliance for Research and Technology, Singapore.

[2] Singapore Immunology Network, Agency for Science, Technology and Research, Singapore.

[3] School of Biological Sciences, Nanyang Technological University, Singapore.

[4] Genome Institute of Singapore, Agency for Science, Technology and Research, Singapore.

[5] Department of Biological Sciences, National University of Singapore, Singapore.

[6] Emerging Infectious Diseases, Duke-NUS Graduate Medical School, Singapore.

[7] Department of Infectious Diseases, Singapore General Hospital, Singapore.

[8] KK Women's and Children's Hospital, Singapore.

[9] Department of Pathogen Molecular Biology, London School of Hygiene and Tropical Medicine, United Kingdom.

[10] Koch Institute for Integrative Cancer Research and Department of Biology, Massachusetts Institute of Technology, Boston, MA 02142, USA.

** Corresponding author: jchen@mit.edu*

Supplementary Figures

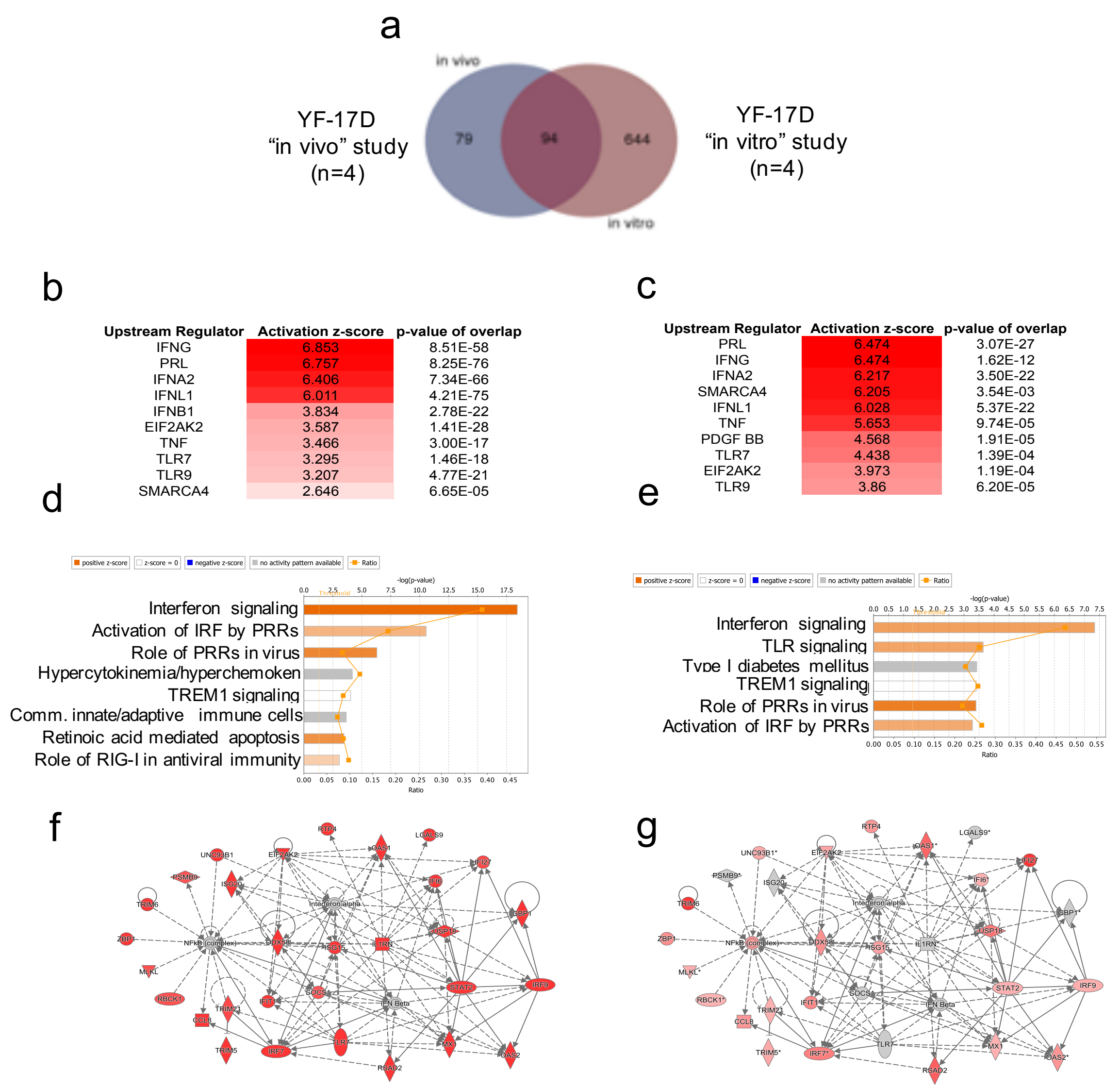

Supplementary Figure 1. Transcriptomic profiles of whole blood from YF-17D vaccinees (*in vivo*) and human PBMCs infected with YF-17D *in vitro*. (a) Venn diagram of individual genes found significantly upregulated by microarray analysis in whole blood of donors vaccinated with YF-17D at 3 days post-vaccination (n=4) and in PBMCs isolated from healthy donors and infected with YF-17D for 48 hours (n=4). (b-g) Ingenuity Pathway Analysis of upstream regulator effects, top pathways activated and top gene networks identified after vaccination (b, d, f) and after PBMC infection (c, e, g).

a

Cell type	Surface markers	Activation markers
mDC-1	Lin1^{-} HLA-DR^{+} CD11c^{+} BDCA-1^{+}	CD80 and CD86
mDC-2	Lin1^{-} HLA-DR^{+} CD11c^{+} BDCA-1^{-}	CD80 and CD86
pDCs	Lin1^{-} HLA-DR^{+} CD123^{+}	CD80 and CD86
Monocytes	CD14^{+} CD16$^{+/-}$	CD54 and CD86
NK cells	Lin2^{-} CD56^{+}	CD69 and NKG2
T helper cells	CD3^{+} CD4^{+}	CD25 and CD69
Cytotoxic T cells	CD3^{+} CD8^{+}	CD25 and CD69
B cells	CD19^{+} CD20^{+}	CD25 and CD69

b

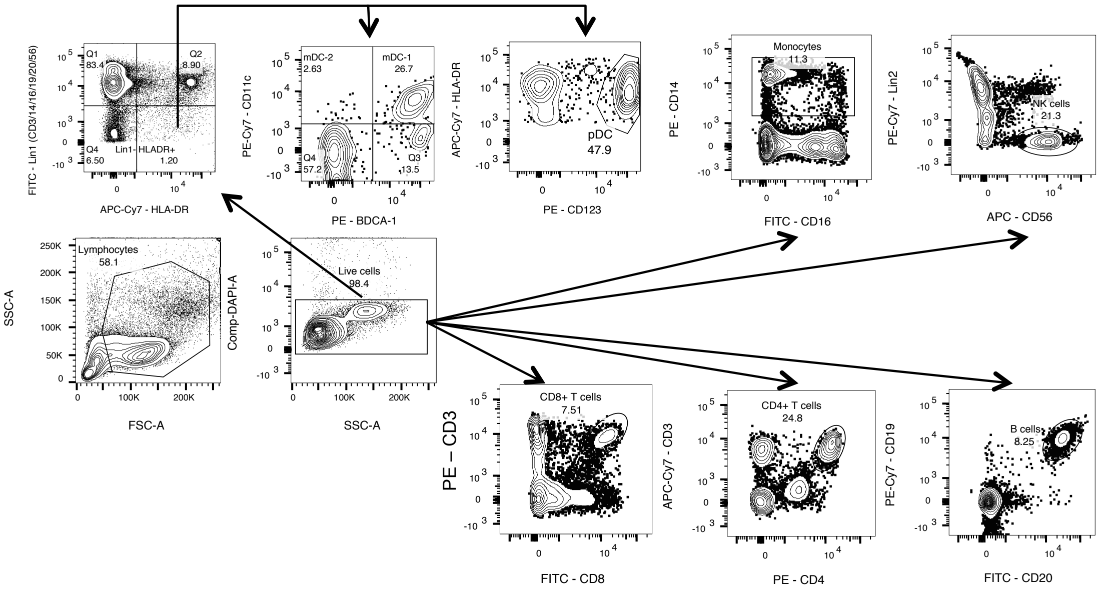

Supplementary Figure 2. Immunophenotyping of infected human PBMCs. (a) Cell type and activation markers used to identify human immune cells in the study. (b) Representative plots and gating strategy.

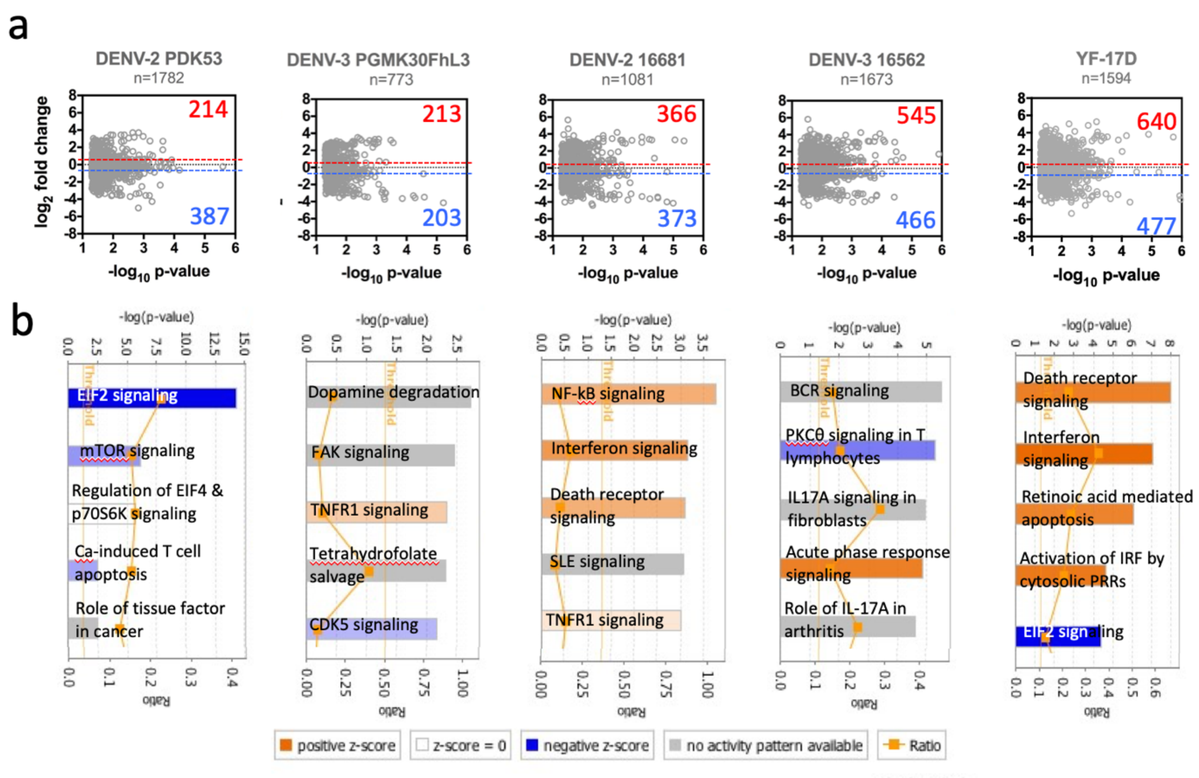

Supplementary Figure 3. Transcriptomic profiles of PBMC responses to flaviviruses. PBMCs from four individual donors were infected at MOI=1 for 48 hours and analyzed by microarray. (a) Volcano plots of all significant changes in gene expression ($p<0.05$) for each virus. The total number of significantly regulated genes is indicated above the graph; the number of genes up- and down-regulated above the threshold of 1.5-fold are indicated in red and blue, respectively. (b) Top 5 IPA pathways identified for each virus when using genes up- and down-regulated above 1.5-fold. The color of the bar denotes the activation Z-score and the length of the bar the log p-value. Blue: down-regulated, grey: no information available for this pathway, orange: up-regulated.

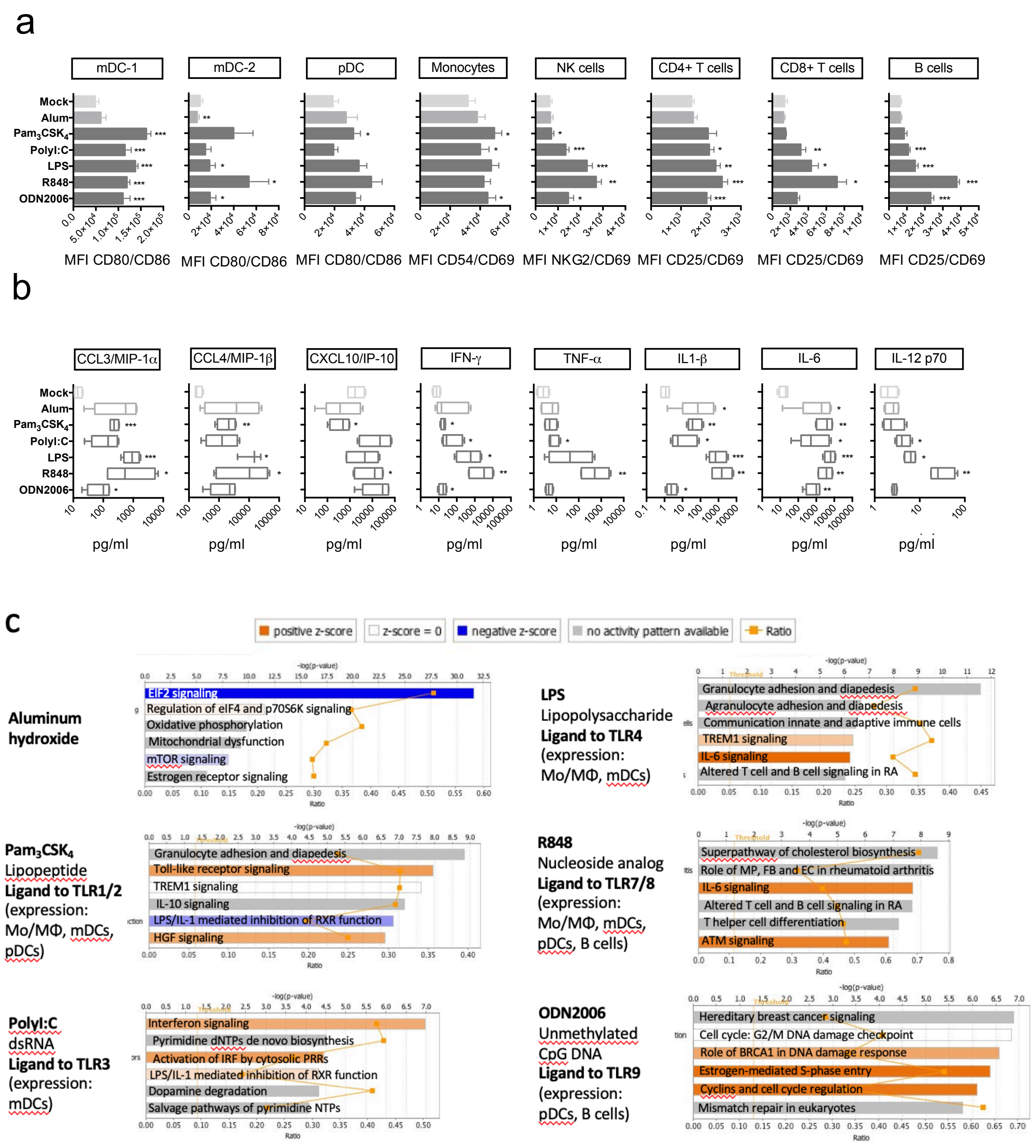

Supplementary Figure 4. Human PBMC response to TLR agonists. PBMCs from four individual donors were stimulated with alum or TLR agonists for 48 hours and analyzed as in Supplementary Figures 1-2. (a) MFI of activation markers on the various immune cells. (b) Levels of cytokines in the culture supernatants. Data shown are box plots depicting the median, 25th and 75th percentiles with whiskers showing the min to max distribution of four independent donors. Significant differences are determined using a two-tailed paired t-test between treated and mock condition for each donor. *p<0.05 **p<0.01 ***p<0.005. (c) Top 5 IPA pathways regulated by each stimulus. For each TLR agonist, the compound category, its receptor and the expected receptor's expression pattern on human immune cells based on literature are indicated.

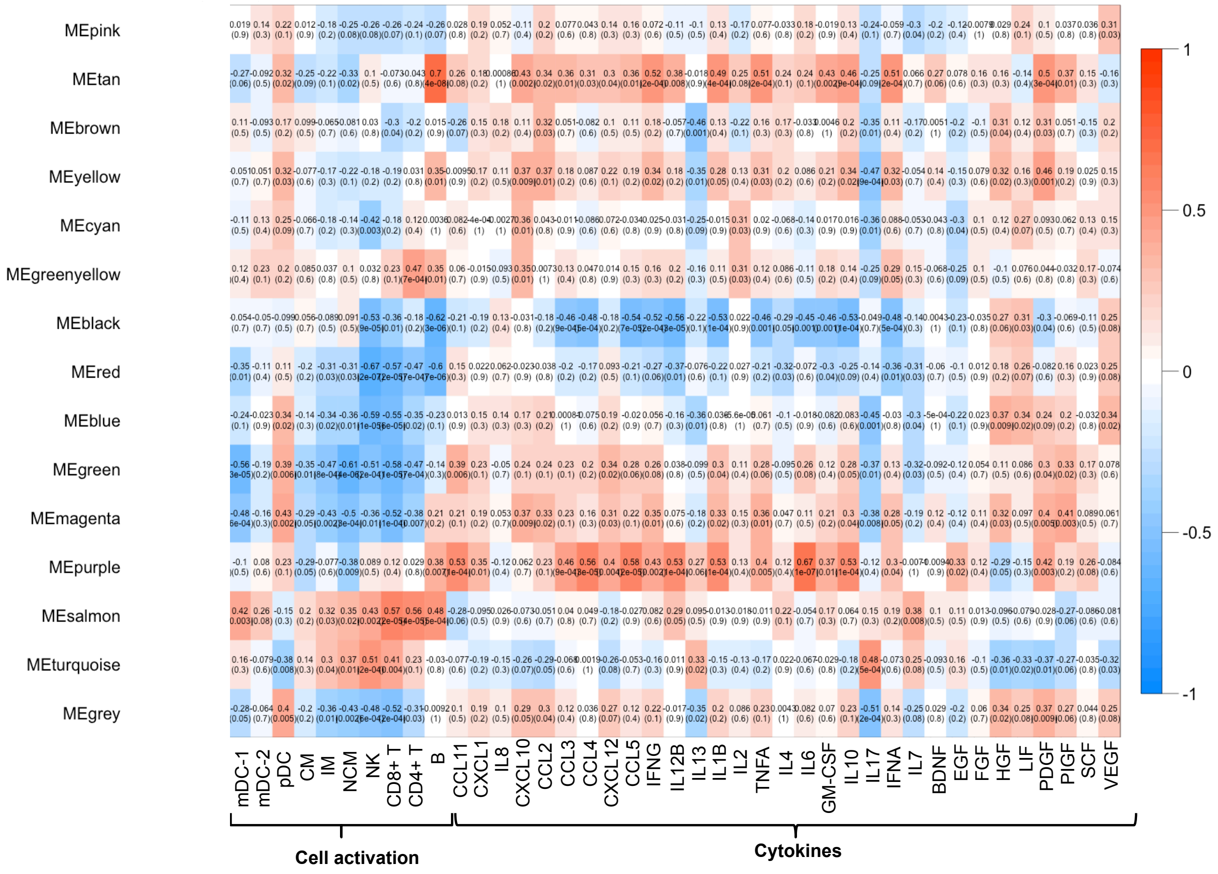

Supplementary Figure 5. WGCNA modules. Correlation matrix between module eigengene and external traits. For each combination the correlation is shown (value between -1 and 1) and the associated p-value is below in parenthesis. Red shows positive correlation and blue negative correlation between the two parameters.

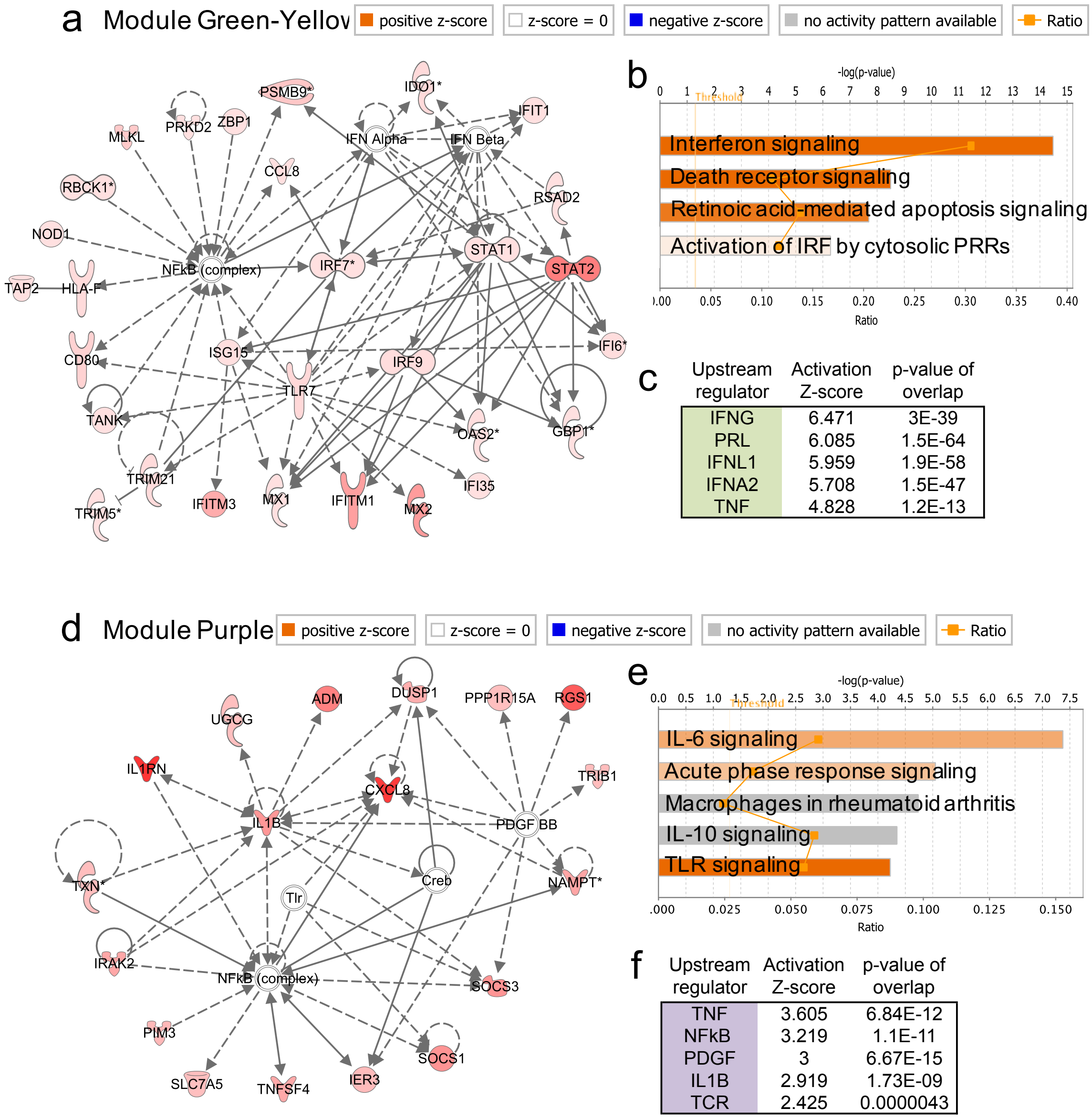

c

Upstream regulator	Activation Z-score	p-value of overlap
IFNG	6.471	3E-39
PRL	6.085	1.5E-64
IFNL1	5.959	1.9E-58
IFNA2	5.708	1.5E-47
TNF	4.828	1.2E-13

f

Upstream regulator	Activation Z-score	p-value of overlap
TNF	3.605	6.84E-12
NFkB	3.219	1.1E-11
PDGF	3	6.67E-15
IL1B	2.919	1.73E-09
TCR	2.425	0.0000043

Supplementary Figure 6. Ingenuity Pathway Analysis of genes in module purple and module green-yellow. (a-c) IPA analysis of transcripts in module Purple. (a) Top gene network, (b) Top 5 pathways and (c) Top 5 upstream regulators. (d-f) IPA analysis of transcripts in module Green-Yellow. (d) Top gene network, (e) Top 4 pathways and (f) Top 5 upstream regulators.

a

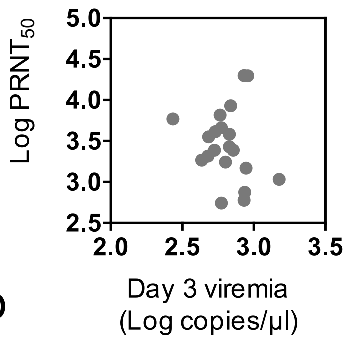

b

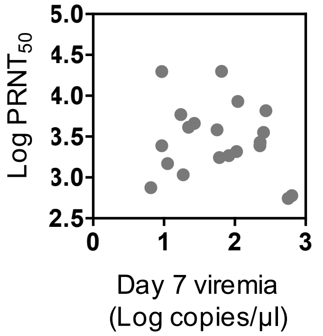

c

Correlation between cytokines and $PRNT_{50}$ in YF-17D vaccinees at 7 days post-vaccination

Module	Cytokine	R^2	p-value
	IL6	0.0004	0.9488
	CCL5/RANTES	0.005	0.8301
	CCL4/MIP-1β	0.153	0.2082
	IL10	0.0001	0.9713
	CCL11/Eotaxin	0.389	0.0303
Purple	IL12p70	0.612	0.0026
	CCL3/MIP-1α	0.312	0.0593
	IFN-γ	0.418	0.023
	PDGF	0.175	0.1754
	TNF-α	0.565	0.0048
	CXCL12/SDF-1α	0.288	0.0719
	CXCL10/IP10	0.321	0.055
Green-Yellow	IL-2	0.432	0.0201
	IFN-α	0.61	0.0027
Blue	VEGF	0.272	0.0822
Blue	HGF	0.272	0.0818
Turquoise	IL17	0.259	0.0912
Salmon	IL7	0.195	0.1513
Magenta	PIGF	0.075	0.3881
Yellow	CCL2/MCP-1	0.124	0.2616
	IL27	0.623	0.0023
Not included in WGCNA analysis	IL15	0.476	0.0131
	IL18	0.381	0.0326
	SCF	0.397	0.0281
	IL22	0.009	0.7711

Supplementary Figure 7. Signatures associated with nAb responses in YF-17D vaccinees. (a-b) Correlation between viremia at day 3 (a) and day 7 (b) after vaccination measured by real-time qRT-PCR and $PRNT_{50}$ titers at one month after vaccination in YF-17D vaccinees. One dot represents one subject in the cohort. (c) List of all cytokines analyzed at 7 days post-vaccination and their correlation with $PRNT_{50}$ titers at one month post-vaccination. Linear regression was performed between the $\log_2$ of the cytokine concentration in the serum and the $\log_{10}$ $PRNT_{50}$. Significantly correlated cytokines (R^2>0.3, p<0.05) are highlighted in red. IL-27, IL-15, IL-18 and SCF, which were not analyzed in the original WGCNA analysis, were also found to correlated with nAb titers in this cohort.

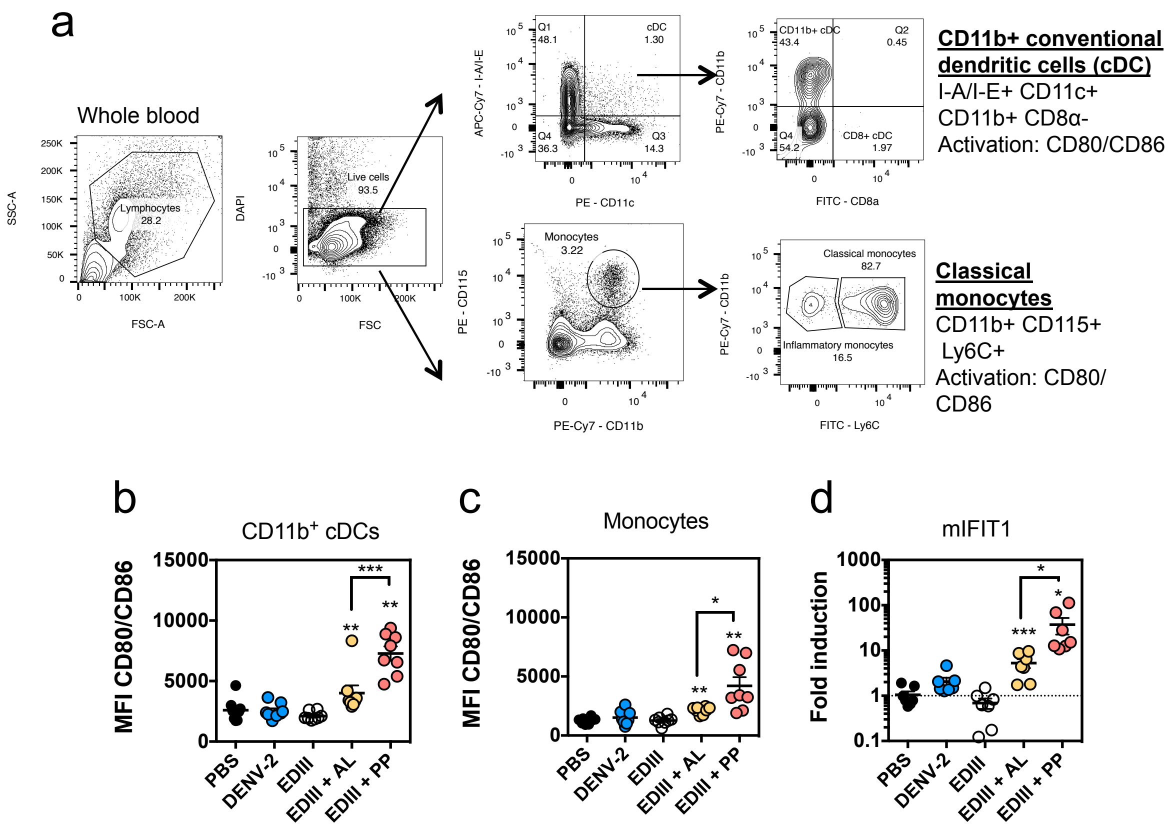

Supplementary Figure 8. PP activation of DC, monocytes and IFN responses in C57BL/6 mice. One day after immunization, blood was taken and analyzed for immune cell activation and induction of IFN responses. (a) Immunophenotyping strategy for identifying conventional CD11b+ dendritic cells (cDCs) and Ly6C+ classical monocytes. (b-c) MFI of activation markers on CD11b+ cDC and Ly6C+ monocytes. The antibodies against both activation markers CD80 and CD86 were labeled with the same fluorophore. (d) Relative levels of IFIT1 transcripts measured by quantitative real-time RT-PCR and normalized to GAPDH. Data shown are the mean ± SEM. Significant differences are measured by unpaired two-tailed t-test (n=8-10 mice per group, from 2 independent experiments). *p<0.05 **p<0.01 ***p<0.005.

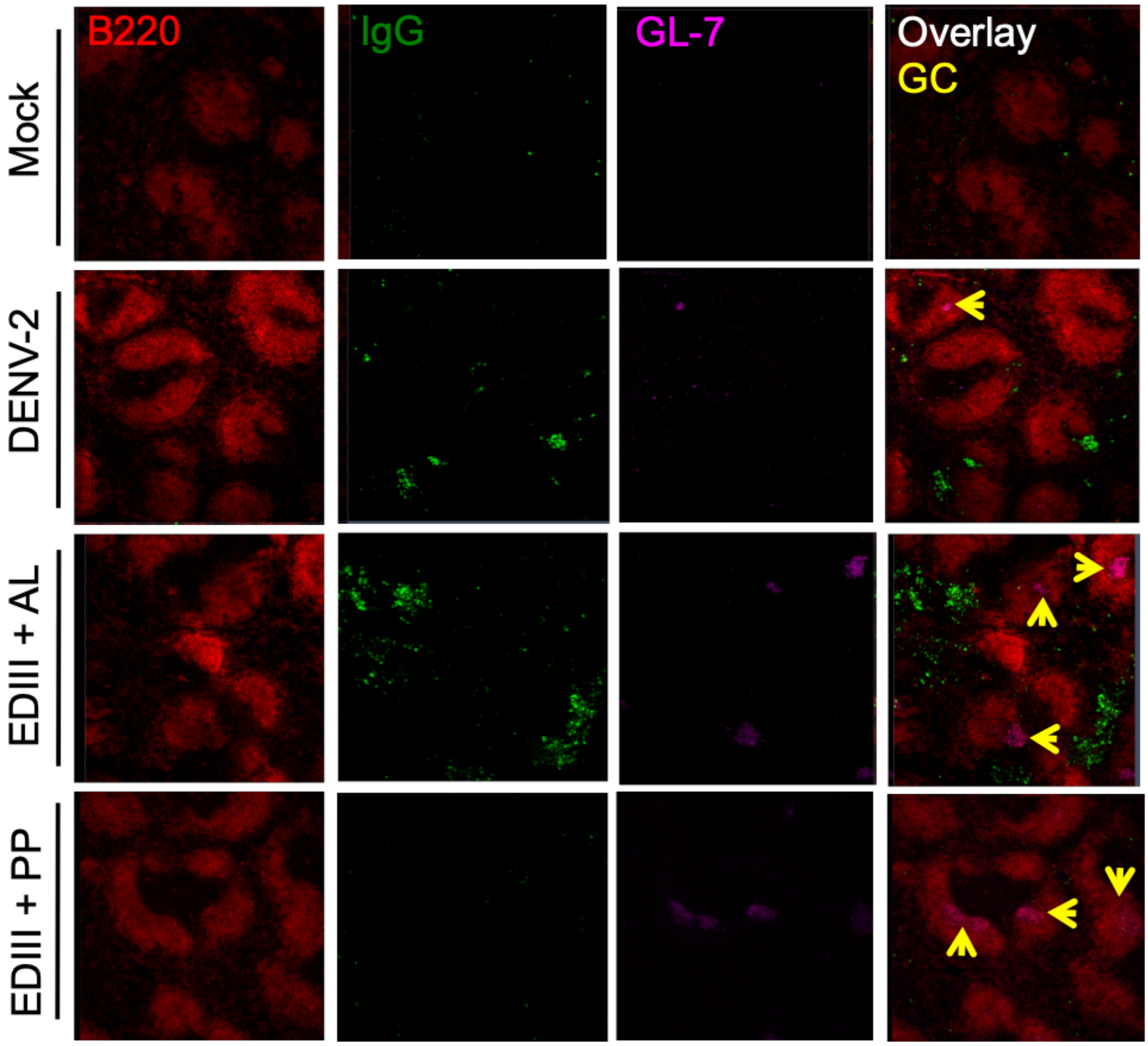

Supplementary Figure 9. Germinal centers in C57BL/6 mice immunized with DENV-2, EDIII + AL and EDIII + PP. Seven days post initial immunization, spleen were harvested from two mice per group and stained for B220 (red), IgG (green) and GL-7 (pink). Representative sections showing IgG, B220 and GL-7 staining alone, or all three overlayed. The colocalization of all three signals indicates a GC (yellow arrows).

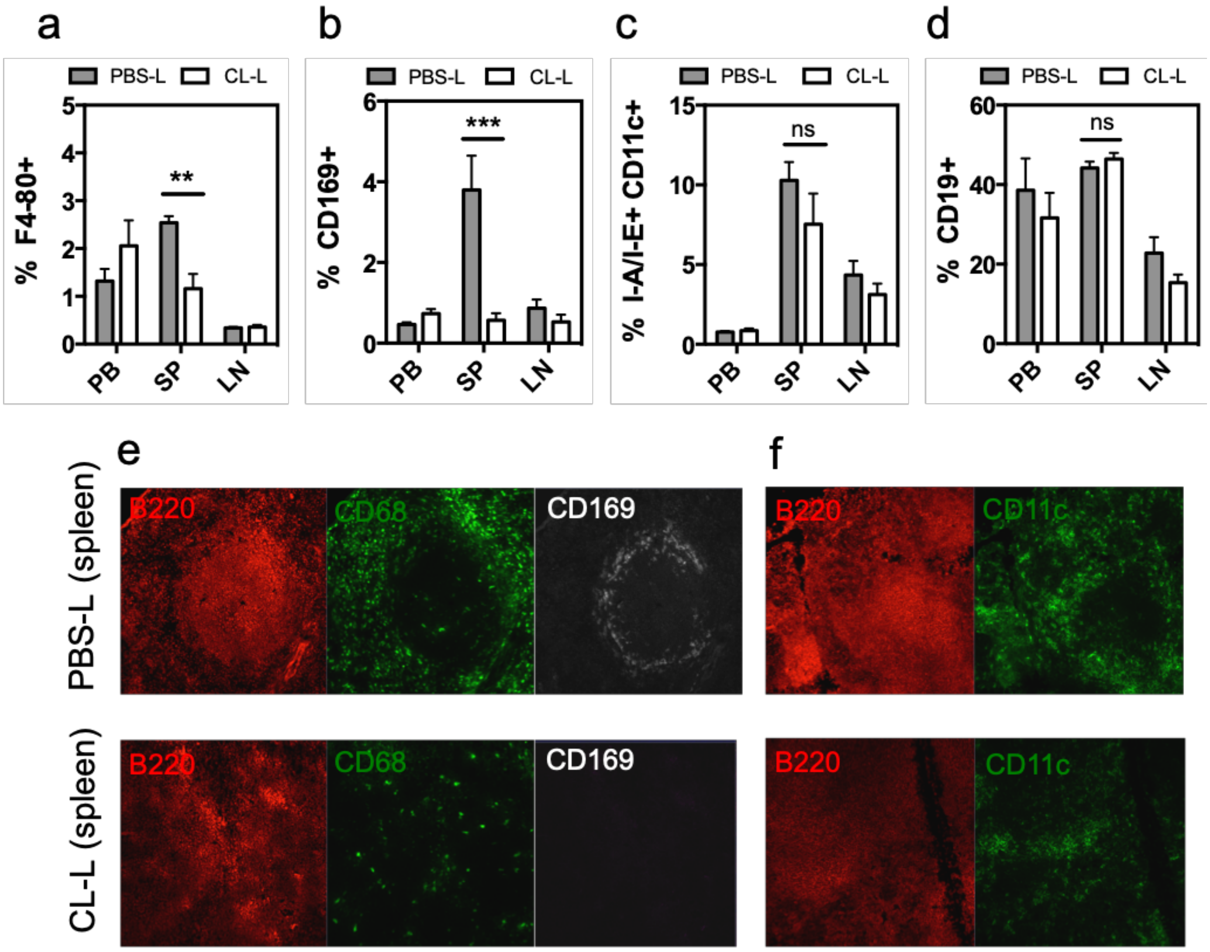

Supplementary Figure 10. MΦs depletion after clodronate liposome (CL-L) or control PBS liposome (PBS-L) treatment in C57BL/6 mice. Mice were injected with CL-L or PBS-L at day -7 and -3 before immunization and a subset of mice sacrificed at day 0 to check for macrophage depletion in different immune compartments. (a-d) Flow cytometry analysis of the frequency of F4/80$^+$ MΦs (a), CD169$^+$ MΦs (b), CD11c$^+$ DCs (c) and CD19$^+$ B cells (d) in the peripheral blood (PB), spleen (SP) and inguinal lymph nodes (LN) (n=4 mice per group from two independent experiments). Data shown are the mean ± SEM. Significant differences are determined by unpaired two-tailed t-test. ns, not significant, **p<0.01 ***p<0.005. (e-f) IFA analysis of spleen sections co-stained with B220 (red), CD68 (green) and CD169 (white) (e) or B220 (red) and CD11c (green) (f).

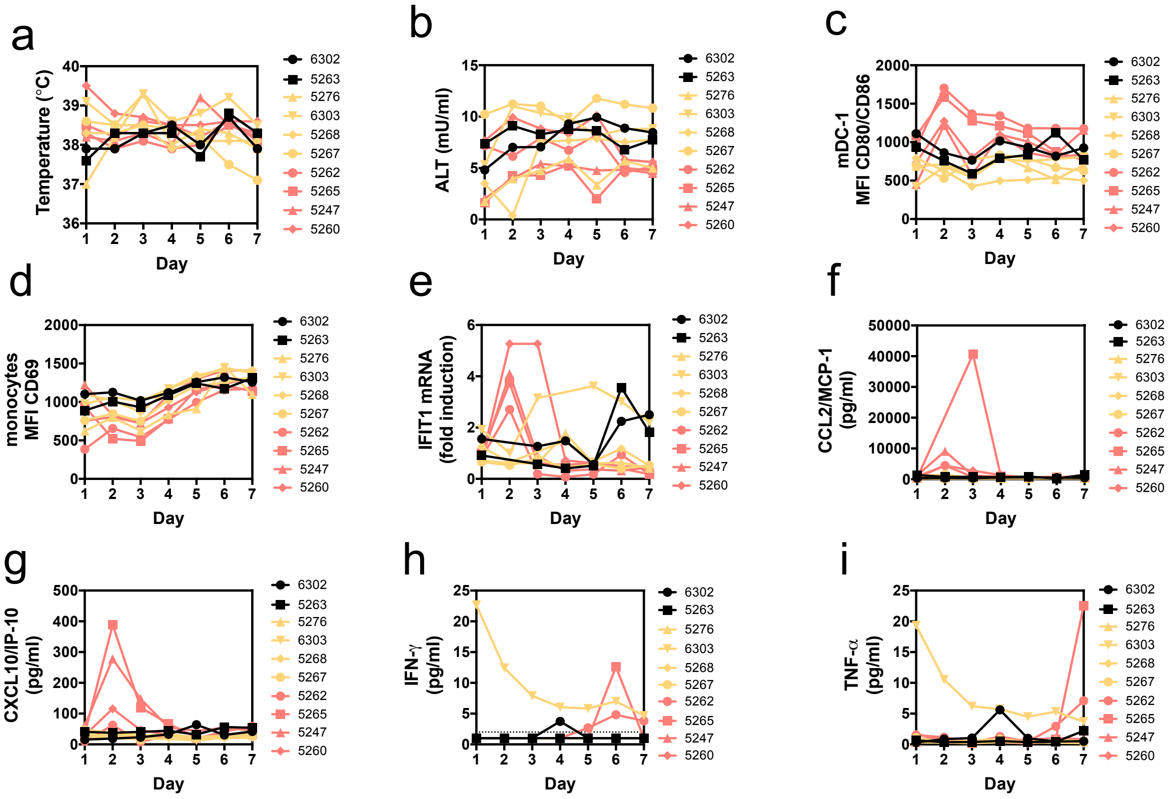

Supplementary Figure 11. NHP response to AL and PP vaccination. Animals were analyzed daily for 7 days after the first vaccination. Results are shown for each individual animal, color-coded by group (black: PBS, orange: EDIII + AL, salmon: EDIII + PP). (a) Rectal temperature. (b) serum ALT enzymatic activity. (c) MFI of activation markers CD80 and CD86 on circulating mDC-1 and (d) MFI of activation marker CD69 on circulating monocytes. (e) Relative levels of IFIT1 transcripts in the whole blood measured by quantitative real-time RT-PCR and normalized to GAPDH. (f-i) serum cytokine levels of CCL2/MCP-1 (f), CXCL10/IP-10 (g), IFN-γ (h) and TNF- α (i).

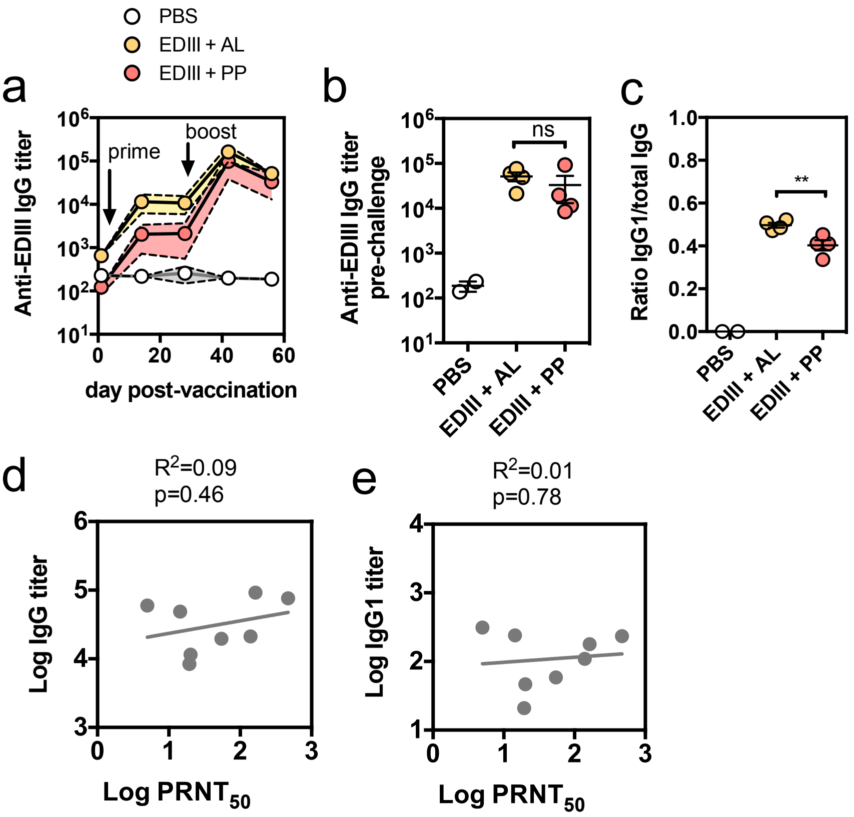

Supplementary Figure 12. NHP Antibody response to PP vaccination. (a) EDIII-specific IgG titers over time. Results are shown by group, with shaded areas representing the mean ± SEM for each group. (b) Log_{10} EDIII-specific IgG titer and (c) Ratio of EDIII-specific IgG1 titer to EDIII-specific IgG titer in the serum of individual animals at day 56 immediately before DENV-2 challenge. Data shown are the mean ± SEM. Significant differences are calculated using an unpaired, two-tailed t-test. ns: not significant **p<0.01. (d-e) Linear regression between total IgG (d) and IgG1 titer (e) and $PRNT_{50}$ titers at day 56. Each dot represents one vaccinated animal.

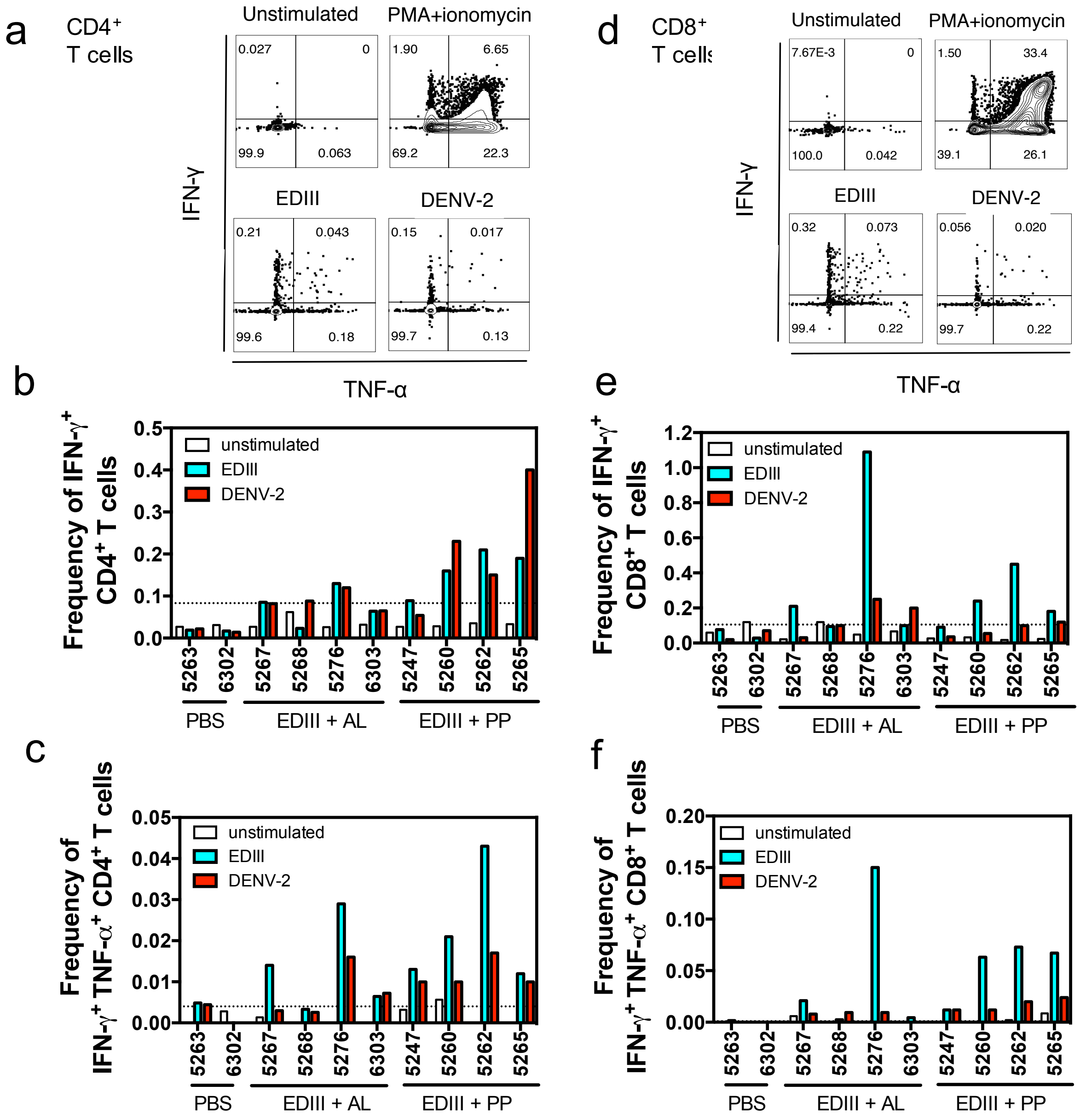

Supplementary Figure 13. NHP T cell response to PP vaccination. Splenocytes were either left unstimulated or stimulated with PMA + iononycine (positive control), EDIII or live DENV-2 virus for 16 hours and analyzed for production of IFN-γ and TNF-α by intracellular staining. (a) Representative TNF-α vs IFN-γ staining profiles of CD4+ T cells from one animal. (b-c) Summary of quantification of IFN-γ^+ $CD4^+$ T cells (b) and IFN-γ^+ TNF-α^+ double positive $CD4^+$ T cells (c) shown as % $CD4^+$ T cells for each animal. (d) Representative TNF-α vs IFN-γ staining profiles of $CD8^+$ T cells from one animal. (e-f) Summary of quantification of IFN-γ^+ $CD8^+$ T cells (e) and IFN-γ^+ TNF-α^+ double positive $CD8^+$ T cells (f) as % $CD8^+$ T cells for each animal. The dotted lines show the threshold from which the response was considered positive, at 3-fold the average frequency of unstimulated cells.

Supplementary Tables

Supplementary Table 1. Viremia in NHP after challenge.

Viremia (PFUs/ml) was measured by plaque assay and viral RNA levels (copies/ml) daily for 7 days after DENV-2 challenge. For each animal identification number, the area under the curve (AUC) between day 57-63 (day 1-7 after challenge) and the peak titer during this time period were calculated with GraphPad Prism.

The lower limit of detection of the assay was <8.5x10^2 copies/ml. nd = not detected

		PBS		EDIII + AL				EDIII + PP			
		**5263**	**6302**	**5267**	**5268**	**5276**	**6303**	**5247**	**5260**	**5262**	**5265**
PFUs/ml	**AUC**	1.6x10^3	2.7x10^3	2.5x10^2	0	1x10^2	0	0	2.5x10^2	0	1.3x10^3
	**Peak**	4.5x10^2	7x10^2	1x10^2	0	0.25x10^2	0	0	0.75x10^2	0	1.3x10^2
copies/ml	**AUC**	1.5x10^6	1.3x10^6	4.4x10^5	7.7x10^4	3.4x10^5	nd	1.1x10^5	2.2x10^5	5.5x10^5	5.9x10^5
	**Peak**	7.7x10^5	6.9x10^5	1.4x10^5	3x10^4	1.3x10^5	nd	5.3x10^4	6.4x10^4	1.7x10^5	1.5x10^5

Supplementary Table 2. Flow cytometry antibody panels for human PBMC phenotyping.

Panel	Antibody	Clone	Reference	Dilution
**mDCs**	FITC lin1		Biolegend 348801	1:100
	APC-Cy7 HLADR	L243	Biolegend 307618	1:100
	PE-Cy7 CD11c	3.9	Biolegend 310608	1:100
	PE BDCA-1	L161	Biolegend 331516	1:100
	APC CD80	2D10	Biolegend 305220	1:100
	APC CD86	IT2.2	Biolegend 305412	1:100
**pDCs**	FITC lin1		Biolegend 348801	1:100
	APC-Cy7 HLADR	L243	Biolegend 307618	1:100
	PE CD123	6H6	Biolegend 306006	1:100
	APC CD80	2D10	Biolegend 305220	1:100
	APC CD86	IT2.2	Biolegend 305412	1:100
**Monocytes**	PE-Cy7 CD19	HIB19	Biolegend 302216	1:100
	PE-Cy7 CD3	HIT3a	Biolegend 300316	1:100
	PE-Cy7 CD20	2H7	Biolegend 302312	1:100
	FITC CD16	3G8	Biolegend 302006	1:100
	PE CD14	HCD14	Biolegend 325606	1:100
	APC CD54	HCD54	Biolegend 322712	1:100
	APC CD69	FN50	Biolegend 310910	1:100
**NK cells**	PE-Cy7 CD19	HIB19	Biolegend 302216	1:100
	PE-Cy7 CD3	HIT3a	Biolegend 300316	1:100
	PE-Cy7 CD20	2H7	Biolegend 302312	1:100
	APC CD56	HCD56	Biolegend 318310	1:100
	PE NKG2	1D11	Biolegend 320806	1:100
	PE CD69	FN50	Biolegend 310906	1:100
**T cells**	APC-Cy7 CD3	HIT3a	Biolegend 300318	1:100
	PE CD4	OKT4	Biolegend 317412	1:100
	FITC CD8	HIT8a	Biolegend 300906	1:100
	APC CD69	FN50	Biolegend 310910	1:100
	APC CD25	BC96	Biolegend 302610	1:100
**B cells**	FITC CD20	2H7	Biolegend 302304	1:100
	PE-Cy7 CD19	HIB19	Biolegend 302216	1:100
	APC CD69	FN50	Biolegend 310910	1:100
	APC CD25	BC96	Biolegend 302610	1:100

Supplementary Table 3. Flow cytometry antibody panel for human MDDCs/ MDMΦs phenotyping.

Antibody	Clone	Reference	Dilution
PerCp-Cy5.5 CD14	HDC14	Biolegend 325622	1:100
APC-Cy7 HLADR	L243	Biolegend 307618	1:100
PE-Cy7 CD11c	3.9	Biolegend 310608	1:100
FITC CD209/DC-SIGN	9E9A8	Biolegend 330103	1:100
PE CD68	Y1/82A	Biolegend 333807	1:100

Supplementary Table 4. Flow cytometry antibody panels for mouse and NHP phenotyping.

Panel	Antibody	Clone	Reference	Dilution
**Mouse mDCs**	APC-Cy7 I-A/I-E	M5/114.15.2	Biolegend 107628	1:100
	PE CD11c	N418	Biolegend 117308	1:100
	PE-Cy7 CD11b	M1/70	Biolegend 101216	1:100
	APC CD80	16-10A1	Biolegend 104714	1:100
	APC CD86	GL-1	Biolegend 105012	1:100
**Mouse monocytes**	PE-Cy7 CD11b	M1/70	Biolegend 101216	1:100
	PE CD115	AFS98	Biolegend 135506	1:100
	FITC Ly6C	HK1.4	Biolegend 306006	1:100
	APC CD80	16-10A1	Biolegend 104714	1:100
	APC CD86	GL-1	Biolegend 105012	1:100
**Mouse MΦ depletion**	FITC F4/80	BM8	Biolegend 123108	1:100
	PE CD11c	N418	Biolegend 117307	1:100
	APC-Cy7 I-A/I-E	M5/114.15.2	Biolegend 107628	1:100
	PerCP-Cy5.5 CD169	3D6.112	Biolegend 142409	1:100
	PE-Cy7 CD19	6D5	Biolegend 115519	1:100
**NHP DCs**	AF488 CD3	SP34-2	BD 557705	1:100
	AF488 CD14	M5E2	Biolegend 301811	1:100
	FITC CD20	2H7	Biolegend 302304	1:100
	APC-Cy7 HLADR	L243	Biolegend 307618	1:100
	APC CD11c	SHCL-3	BD 333144	1:100
	PE BDCA-1	AD5-8E7	Miltenyi 130-113-864	1:50
	PerCp-Cy5.5 CD123	7G3	BD 558714	1:100
	PE-Cy7 CD80	L307.4	BD 561135	1:100
	PE-Cy7 CD86	2331	BD 561128	1:100
**NHP monocytes**	APC-Cy7 CD3	SP34-2	BD 557757	1:100
	APC-H7 CD20	2H7	BD 556633	1:100
	PerCp-Cy5.5 CD14	M5E5	BD 550787	1:100
	FITC CD16	3G8	BD 555406	1:100
	PE-Cy7 CD86	2331	BD 561128	1:100
	APC CD69	FN50	Biolegend 310910	1:100

Supplementary Table 5. Primers used in the study.

**Mouse**	**mIFIT1**	5’ AGCTTCCATGTGAAGTGACATCT 3’<br>5’ CAAGGCAGGTTTCTGAGGAG 3’
	**mGAPDH**	5’ TTGATGGCAACAATCTCCAC 3’<br>5’ CGTCCCGTAGACAAAATGGT 3’
**NHP**	**cmIFIT1**	5’ CAGAATGAGGAAGCCCTGAAGAGC 3’<br>5’ CCTTGTCCAGGTAAGCCTGGG 3’
	**cmGAPDH**	5’ GGACTCATGACCACAGTCCACG 3’<br>5’ GAGCTTCCCGTTCAGCTCAGG 3’

Supplementary Table 6. Flow cytometry antibody panel for NHP T cell responses.

Antibody	Clone	Reference	Dilution
Near IR Live/Dead		Invitrogen L10119	1:500
AF488 CD3	SP34-2	BD 557705	1:100
PerCP-Cy5.5 CD4	L200	BD 552838	1:100
PE CD8	RPA-T8	BD 557086	1:100
PE-Cy7 IFN-γ	B27	Biolegend 506518	1:100
APC TNF-α	MAB11	Biolegend 502912	1:100
